# Consumer Behaviour through the Eyes of Neurophysiological Measures: State-of-the-Art and Future Trends

**DOI:** 10.1155/2019/1976847

**Published:** 2019-09-18

**Authors:** Patrizia Cherubino, Ana C. Martinez-Levy, Myriam Caratù, Giulia Cartocci, Gianluca Di Flumeri, Enrica Modica, Dario Rossi, Marco Mancini, Arianna Trettel

**Affiliations:** ^1^Department of Molecular Medicine, Sapienza University of Rome, Viale Regina Elena, 291, 00161 Rome, Italy; ^2^BrainSigns Srl, Via Sesto Celere 7/c, 00152 Rome, Italy; ^3^Department of Communication and Social Research, Sapienza University of Rome, Via Salaria, 113, 00198 Rome, Italy; ^4^Department of Anatomical, Histological, Forensic & Orthopedic Sciences, Sapienza University of Rome, Piazzale Aldo Moro 5, 00185 Rome, Italy

## Abstract

The new technological advances achieved during the last decade allowed the scientific community to investigate and employ neurophysiological measures not only for research purposes but also for the study of human behaviour in real and daily life situations. The aim of this review is to understand how and whether neuroscientific technologies can be effectively employed to better understand the human behaviour in real decision-making contexts. To do so, firstly, we will describe the historical development of neuromarketing and its main applications in assessing the sensory perceptions of some marketing and advertising stimuli. Then, we will describe the main neuroscientific tools available for such kind of investigations (e.g., measuring the cerebral electrical or hemodynamic activity, the eye movements, and the psychometric responses). Also, this review will present different brain measurement techniques, along with their pros and cons, and the main cerebral indexes linked to the specific mental states of interest (used in most of the neuromarketing research). Such indexes have been supported by adequate validations from the scientific community and are largely employed in neuromarketing research. This review will also discuss a series of papers that present different neuromarketing applications, such us in-store choices and retail, services, pricing, brand perception, web usability, neuropolitics, evaluation of the food and wine taste, and aesthetic perception of artworks. Furthermore, this work will face the ethical issues arisen on the use of these tools for the evaluation of the human behaviour during decision-making tasks. In conclusion, the main challenges that neuromarketing is going to face, as well as future directions and possible scenarios that could be derived by the use of neuroscience in the marketing field, will be identified and discussed.

## 1. Introduction

In the last years, we have had a growing interest in the use of brain imaging techniques, for the analysis of brain responses to different contexts. Scientific development in recent years was characterized by an expansion in the application of different and multidisciplinary research modalities to answer various questions of a given scientific field. The recent “*boom*” in employing neuroscientific methods to better understand human behaviour in various contexts is undoubtedly interesting and intriguing. Tallis [1] coined the term “*Neuromania*” to refer to the embracing of neuroimaging by various fields of studies to explain all human phenomena in terms of brain activity.

Because of the potential application of neuroscientific methodologies to better understand unconscious reasons of human behaviours, especially in terms of risky behaviours, the interest on such approaches initially focused on the investigation of human factors (HFs) [2]. In fact, this kind of application relies on specific social challenges, such as operational environments where safety of people relies on the work and the efficiency of one or more operators. For example, in the transports' domain, the passengers' safety depends on the performance of the pilot(s) [3–8], of the air-traffic controller(s) [9–14], or of the driver(s) [15–17]. In such contexts, a human error could have serious and dramatic consequences.

In particular, HFs have consistently been identified to be the main responsible factor, in a high proportion, of all workplace accidents. It has been estimated that up to 90% of accidents exhibit HFs as a principal cause [18]. Consequently, the HF construct is receiving more and more attention and has been investigated across a wide range of domains. In many operational environments (e.g., aircraft piloting, air-traffic control, industrial process control, and robot-assisted surgery), operators have to constantly manage complex systems and machines to accomplish operational activities. Improvements in such technologies or even new solutions are often proposed, with the aim to enhance security and efficiency in the human-machine interaction (HMI) and consequently to increase the operator's performance and thus overall safety [19, 20]. In this context, the most studied user's mental state is the mental workload, e.g., the level of cognitive demand induced by a task [21], due to its strong relationship with the user's performance variations [9], but also other mental states such as vigilance, situation awareness, stress, drowsiness, and mental engagement received attention from the scientific community [22–25].

However, until the last decade, these kinds of applications were still seen as pure research, far from being reproducible on large scale outside the laboratory and related to everyday activities.

Nevertheless, thanks to the technological progress and the development of innovative solutions applied to neuroimaging, such as less invasive and wearable devices [26, 27], the neuroscientific approach became a powerful tool to investigate unconscious reactions and the brain functioning during daily life. In other words, it investigates how the human being perceives, processes, evaluates, reacts, and utilizes the external stimuli in the decision-making process in everyday activities and interactions [28].

In this context, *industrial neuroscience* is a new emerging area in which state-of-the-art methodologies are applied in real contexts to evaluate the cognitive and emotional states of humans [29].

Within the last 10 years, the economic world approached those new techniques, leading neuroscience labs to address problems and questions regarding economic transactions. In this framework, neuroscience researchers and economists have to cooperate on the evaluation of brain activity related to economic value judgments and to the understanding of the underlying mechanisms of decision-making processes in real-world settings [30].

This cooperation has given rise to a new area of study called “*neuroeconomics*” that uses all the modern tools of neuroimaging [31–37].

One of the biggest questions in today's market is what drives consumers to decide on one product instead of another, or why consumers interact with a specific brand. So, there is a growing interest in understanding how brain responses reflect the decision-making process of consumers. In this regard, the practical use of neuroimaging neuroscientific tools in real contexts and for real stimuli, i.e., the subject of this article, is named in the literature “*neuromarketing*.” The term specifically describes a field of study defined as “*the application of neuroscientific methods to analyse and understand human behaviour in relation to markets and marketing exchanges*” [38]. This definition has two main upshots: (1) it moves consideration of neuromarketing away from being solely the use of neuroimaging by commercial interests for their benefit and (2) the scope of neuromarketing research is widened by solely consumer behaviour to include many more avenues of interest, such as inter- and intraorganizational research, which is common in the marketing research literature.

Neuromarketing studies seek to investigate different brain areas while experiencing marketing stimuli to find and report the relationship between customer behaviour and the neurophysiological system. Using knowledge and know-how from human brain anatomy, and knowing the physiological functions of brain areas, it is possible to model neuronal activity underlying specific human behaviours. Through neuroimaging methods, researchers can compare different brain area activations during a specific task, in order to develop models which can not only describe the dynamics of human decisions but also understand the usual mismatches between consumers' thoughts and their actions [39–43].

This paper describes the state-of-the-art of the discipline of neuromarketing and provides a review of the main studies conducted in the area in the last two decades. Thus, this paper presents a better understanding of neuroscientific technologies and how these can be employed to study the human behaviour in real contexts. This review discusses the ethical issues linked to the use of the neuroscientific tools for the evaluation of the human behaviour during purchases. Finally, future research avenues and possible scenarios are considered.

## 2. History of Neuromarketing

For hundreds of years, people tried to understand how we make—or should make—a decision. The question kept alive some disciplines, such as philosophy and psychology. Decades of research have shown that much of our mental processing occurs at the subconscious level, including the decision that we take as consumers. These subconscious processes explain why we fail so often to accurately predict our own future choice [44]. Often, what we think we want has little or no bearing on the choices we actually make [45].

“*Consumer neuroscience*” is a new approach within consumer research that has rapidly developed, which aims to enhance the understanding of consumer behaviour using insights and methods from neuroscience.

The birth of the field of consumer neuroscience has generated wide-ranging, ongoing debates of whether this hybrid field benefits its parent disciplines (consumer psychology and neuroscience) and, within them, what forms these benefits might take [38, 46–48]. In order to appreciate the value of a combination of neuroscience with consumer psychology, it is important to understand the broad range of insights available from cognitive neuroscience.

Cognitive neuroscience is the study of the nervous system that seeks to understand the biological basis of behaviour. In cognitive neuroscience, the main distinction is between clinical and nonclinical research. The former, known as neurology, studies the patients and how nervous system disorders, trauma, tumours, and injuries affect their cognition, emotion, and behaviour as compared to healthy subject populations. The second one studies consumer responses in healthy subject populations. A last critical distinction is between consumer neuroscience that, as previously stated, refers to academic research at the intersection of neuroscience and consumer psychology and “*neuromarketing*,” which refers to the application of consumer neuroscience in the marketplace using neurophysiological tools, such as eye tracking, electroencephalography, and functional magnetic resonance imaging, to conduct specific market research. Indeed, neuromarketing can be defined as “*the field of study that applies the methodologies of the neuroscience to analyse and understand the human behaviour related market and economic exchanges*” [38]. Hence, neuromarketing is related to marketing as neuropsychology is related to psychology. Additionally, neuropsychology studies the relationship between the human brain and cognitive and psychological functions, while neuromarketing investigates consumer behaviour from a brain perspective [49].

Even if the term “neuromarketing” cannot be attributed precisely to anybody, Professor Ale Smidts of the Rotterdam School of Management of the Erasmus University is known as the first one who used the term “*neuromarketing*” that refers to the use of neuroscientific techniques by the marketing discipline in 2002 [50]. At the time, two US companies—BrightHouse and SalesBrain—became the first ones to offer neuromarketing research and consulting services, promoting the use of technology and knowledge coming from the field of cognitive neuroscience in the business field. Specifically, Atlanta-based BrightHouse announced the creation of a department dedicated to fMRI for marketing research purposes [51, 52]. This demonstrates that even before this scientific approach was provided with the prefix “*neuro*,” some companies were already using neurophysiologic techniques, such as EEG, to solve marketing problems [53–57].

Hence, the contribution of neuroscientific methods became significant for the knowledge on the human behaviour in the marketing scope. Recent years have seen a rise in the abilities of neuroscientists to study the brain activity, and in this field, the contribution of neuromarketing has been very useful to answer several questions about which consumers' neural processes are involved in correspondence of behavioural performance and at different levels of consumer research. Moreover, another interesting issue is overcoming the dependence on the verbal answers that nowadays are used for testing subjects in traditional marketing research studies, where insights and indicators depend on the good faith and accuracy of the experimental subject reporting his own sensations and opinion to the experimenter. Indeed, traditional data collection methods have some limitations and are criticized for not revealing accurate results. Some studies set the failure rate of new products at 90% [58–60]. This gives clues that traditional marketing studies conducted prior to the launch of the products do not produce reliable, valid, and generalizable results.

The traditional techniques allow to measure the cognitive and emotional experiences only as verbally expressed at the conscious level during the interview. Instead, by using brain imaging techniques, it is possible to distinguish the unconscious states related to processes that have a key role in influencing behaviours, integrating what can be found by verbal or written declarations.

Interestingly, some experimental evidence suggests that the use of the brain imaging, in the near future, could be placed side by side with classical tests that are mainly used today in the marketing science [61].

Therefore, from the marketing point of view, neuromarketing is an important and revolutionary field of marketing research, and also defined as the “*third dimension*” of it. Because of the reasons mentioned above, neuromarketing has received considerable attention in the corporate world, and the growth of neuromarketing companies in the recent period has been impressive [62].

**Figure 1:**
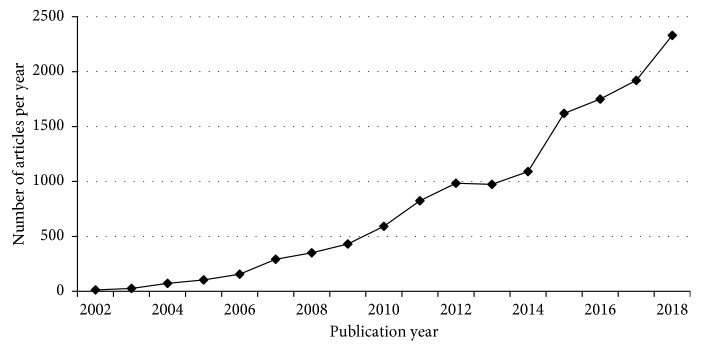
Evolution of academic interest in neuromarketing (as in the number of articles published on the topic) from 2002 to 2018 (source: http://www.scholar.google.com).

Moreover, during the last decade, the number of publications in a top marketing journal and Google references concerning this topic has grown exponentially (Figure 1), and the same holds for the number of neuromarketing companies founded.

The total number of neuromarketing papers published so far is 16500 (source: Google Scholar in March 2019). In 2008, Hubert and Kenning [63] reported more than 800,000 Google hits for the term “*neuromarketing*,” and in 2012, the same search yielded over 1.4 million hits, underlining the rising interest in this topic. In 2018, there have been more than 3 million hits for the term “*neuromarketing*.”

**Figure 2:**
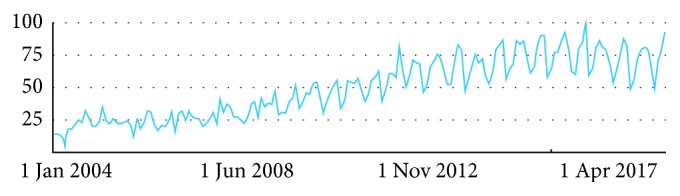
Evolution of general and global interest in neuromarketing since 2004 until recent years (source: https://trends.google.com/trends/).

Nowadays, companies around the world that offer neuromarketing research services are growing. Since neuromarketing arrived in the public consciousness levels, it received a tidal wave of enthusiasm, which is ever growing. In Figure 2, the neuromarketing interest growth in the world, in terms of entering the word “*neuromarketing*” in the Google search portal, can be seen.

Given the growing dimension of the neuromarketing phenomenon, the Neuromarketing Science and Business Association (NMSBA; http://www.nmsba.com), an international organization for the coordination of the activities in this new field of market research, was funded in 2012. Its main aim is to diffuse the best practice in the field of neuromarketing and to connect the major companies that offer such services across the world. Today, company members of the NMSBA are found across 42 countries (NMSBA, 2019). The highest concentration of vendors is in Europe (54) and Central and South America (27). Other vendors are located around the globe in Asia (13), North America (11), Middle East (3), and Africa (1). By country, the United States and United Kingdom have the most of members (10 each), followed by the Netherlands (9), Italy and Germany (6 each), and Spain and Turkey (5 each) (https://bit.ly/2JIwmzu). Importantly, neuromarketing today has expanded to the point where buyers can find nearby vendors with global or local expertise in almost every region of the world (https://bit.ly/2UbeWzz).

## 3. The Added Value of the Neuroscience Techniques

Which is the added value of using neuroelectrical brain imaging tools for marketers? Neuromarketing techniques are commonly used in communication and advertising areas. The use of these technologies makes possible to identify advertising elements that trigger positive feelings [64–66] and which are the features that should not be present in communication as they may cause consumer aversion to the products. They also allow to select visual and audio features, as well as the timing and selection of appropriate media [65]. Neuromarketing in addition has the ability to identify consumers' needs and, therefore, to develop more useful and pleasant products [67]. The contribution of neuromarketing also helps enhancing branding or brand positioning strategies. Moreover, neuromarketing has the capability to adjust strategies of pricing and product development, as demonstrated by several researchers [38, 52, 64, 68]. Neuromarketing has an enormous potential to identify causes of purchasing disorders such as compulsivity [51, 64, 65, 69] and to develop more effective social campaigns, such as encouragement for the use of seat belts in cars [70] and for antismoking campaigns [71–75].

Indeed, by neuromarketing, the strength of emotional attachments to a brand and the instinctive impact of stimuli to be implemented on a point of sale to encourage purchases were also evaluated and studied [76, 77]. This list can be extensive and applied to each specific marketing area, as required by marketing management.

Modern consumers are different from the past and, surely, different from how the future ones will be. In the same way, the present marketplace is fundamentally different because of major societal forces that have resulted in many new consumers and company capabilities. These forces have created new opportunities and challenges and changed marketing management significantly, as companies constantly seek new ways to achieve marketing excellence. Learning about consumers is the key to implementing the marketing concept and exercising marketing imagination.

The American Marketing Association defines consumer behaviour as “*the dynamic interaction of affect and cognition, behaviour, and the environment by which human beings conduct the exchange aspects of their lives*” [78]. Accordingly, this definition includes the thoughts and feelings that people experience and the actions they perform in consumption processes. Thus, it involves all aspects of the environment that can influence human thoughts, feelings, and actions, including opinions from other consumers. For instance, products and packaging, brands, advertisements, price information, and many other aspects can be considered environmental factors. Indeed, consumer behaviour is a complex phenomenon for investigation and thus is a heterogeneous field. Marketing academics have published research papers mostly about consumer behaviour who have a multidisciplinary skill about training, objectives, and methods.

Advances in neuroimaging technology have led to an explosion in the number of research studies studying the living human brain, thereby developing the understanding of its structures and functions. With the explosion of impressive images from brain scans in both scientific and popular media, researchers from other fields in the social and behavioural sciences naturally become interested in the application of neuroimaging to their own research. The possibility to “*get inside the heads*” of customers has aroused an increase of interest in commercial enterprises for discovering the real needs of them and proposing the products or services that meet their specific desires and needs. But it is very important to highlight that, with neuromarketing techniques, companies have “*just*” the opportunity to better understand the consumer behaviour and which are the processes underlying the decision-making process. Accordingly, this does not constitute the “*buy button*” to induce to buy products or services which companies promote.

With the help of advanced techniques of neurology, which are applied in the field of consumer neuroscience, a more direct view into the “*black box*” of a consumer should be possible. Consumer neuroscience should not be perceived as a challenge to traditional consumer research but constitutes a complementing advancement for further investigation of specific decision-making behaviour; as mentioned above, it could be defined as the third dimension of it (after the qualitative and the quantitative marketing research). In such a scenario, brain imaging techniques, applied to human decision-making mechanisms, could be adopted to corroborate results obtained by traditional techniques.

Daniel Kahneman, who in 2002 won the Nobel Prize for integrating advances in psychological research to economic science, analysed the complexity of people's reasoning when making economic decisions and demonstrated that when people choose, they do not always do it objectively. In his book, *Thinking, Fast and Slow*, he described how different systems of thought can affect judgment when people make decisions. The distinction between “*fast*” and “*slow*” thinking has been explored by many psychologists over the last 25 years. Kahneman did not invent the System 1-System 2 model of the brain processes, but his work over the last several decades has popularized it as one of the most useful overarching frameworks for understanding how the human brain works and, in particular, how the unconscious and conscious parts of the mind work together. System 1 and System 2 are neutral terms describing two distinct sensory processing and decision-making systems in the brain. System 1 is fast, automatic, and outside our volitional control; System 2 is slow, voluntary, and under our control [79]. This model is the key to understand why traditional research as interviews, focus groups, and surveys is at risk of bias and why neuromarketing has emerged as an alternative and integrator to them. Traditional marketing research was based on a System 2 view of the brain, assuming that consumers have always access to their mental states and that they can accurately describe what they want and why they choose products and/or services. Instead, neuromarketing has emerged because, through neurometric tools, scientists can offer new research methods that can also measure System 1 processes and provide new insights to understand how and why consumers respond to marketing stimuli and interact in the marketplace [79].

In addition, we know from cognitive psychology that emotions too play an important role in memory processes: emotions can help us learn and remember [80]. Consumers are no longer considered completely rational because emotion and unconscious and automatic processes play a central role in generating behaviour [81, 82]. Therefore, these previous investigations showed that humans are not perfectly rational in making decisions.

Therefore, the challenge lies on how to use the neuroscientific tools to discover the brain or physiological instinctive reactions to take into account their effects and to define the best strategies to reach better the consumer needs. In such a way, neuromarketing is an exciting promise for marketing evolution in the future and at present.

Nowadays, the most part of neuroimaging studies are conducted in specialized institutes, and most of the largest marketing research companies and advertising agencies have neuromarketing divisions (e.g., Nielsen, Ipsos, and Millward Brown) with clients that represent an impressive list of brands across a variety of product categories (e.g., Google, Campbell's, Estée Lauder, and Fox News) [83].

Nevertheless, the use of neuromarketing activities has aroused some disagreements because critics of the subject believe that the use of such techniques would affect consumers' ability to avoid marketed products, leaving the individuals unable to resist such efforts and making them easy targets for the company's campaigns [84]. Some researchers furthermore believe neuromarketing to be science fiction rather than reality-based science, given that thoughts are individual and strictly dependent on personal experiences and character, which makes virtually and practically impossible to find people with identical thoughts [85]. Supporters of neuromarketing, such as Lindstrom [86, 87] and Dooley [88], on the contrary, discuss the benefits of neuromarketing techniques for both consumers and organizations; they suggest that tailored products and campaigns benefit consumers by facilitating their decisions instead of manipulating them. At the same time, organizations can ensure greater competitiveness by saving a large portion of their budgets currently spent on inefficient and ineffective marketing campaigns.

## 4. Brain Imaging Tools in Marketing Research

The possibility to acquire signals and images (neuroimaging) from the human body has become vital for early diagnosis [89], not only for marketing research [90] but also for human-machine interaction studies [91, 92], automation, and system design [93, 94]. It is possible to obtain these data in the form of electrobiological signals, for example, from the heart by an electrocardiogram (ECG), from muscles by an electromyogram (EMG), from the brain by an electroencephalogram (EEG) and magnetoencephalogram (MEG), from the stomach by an electrogastrogram (EGG), and finally from eye nerves by an electrooculogram (EOG). Measurement can also have the form of one type of ultrasound or radiograph such as sonograph (or ultrasound image), computerized tomography (CT), magnetic resonance imaging (MRI) or functional MRI (fMRI), positron emission tomography (PET), and single-photon emission tomography (SPET) [95].

As previously seen, psychophysiological techniques have been applied since the 1960s in consumer research to measure pupillary dilation (through the eye tracker) and electrodermal response (through the heart rate) [96]. Similarly, EEG started to be used in such studies in the early 1970s, specifically while a viewer watched television [97]. Subsequently, several researchers followed up on this work [54, 56, 98].

The first official study on neuromarketing was conducted in 2003 and was published on neuron in 2004 by Read Montague's team [99] which used a brand experiment to demonstrate the dominance of the frontal lobe (specialized in executive function) over the limbic system (responsible for emotional and instinctual behaviour) in the product choice. Specifically, in this study, they used fMRI to find correlates of people's preferences for two similar sugared drinks: Coke and Pepsi. The study involved 67 participants divided into four groups; each group was given a separate taste test outside the scanner and a drink delivery paradigm while they were instead inside the scanner. Before conducting the taste test, participants were asked which drink they preferred to consume between Coke and Pepsi, or whether they had no preference between them. The study findings highlighted that different parts of the brain are active if people are aware or not of the proposed brand, and in such a case, a strong brand such as Coca-Cola has the power to “*own*” a piece of our frontal cortex [49]. The Coke and Pepsi study results had many people worried about their potential power because of the fear that they harboured a hidden code to tweak our perceptions below the level of our consciousness. About ethical questions, in 2004, the journal *Nature Neuroscience* published an article entitled “*Brain Scam*” about this issue behind neuromarketing studies questioning the morality of neuromarketers. In response, Dr. Michael Brammer, the CEO of Neurosense, a company that was mentioned in this article, eloquently replied to the editor of the journal agreeing to be careful in the exploitation of any new technology but that the scientific rigour and ethical issue must apply to all scientist activities.

Notably, this short-lived attack from the media did not dissuade Harper Collins from adding the word “*neuromarketing*” to his dictionary in 2005. And by 2006, neither the critical article from *Nature Neuroscience* nor the efforts deployed by the consumer advocacy group Commercial Alert succeeded in curbing the popularity and growth of neuromarketing.

Thanks to the progress and the new development of the technology, the modern neuroscientific tools are always multifaceted and versatile and in the last years have deeply advanced improving spatial and temporal resolution and also improved in their size with the development of some technologies with more wearable, ergonomic devices. So it is easier to investigate the brain functioning: how the human being perceives, processes, evaluates, reacts, and utilizes the personal variation in decision-making in everyday interactions, not only in the laboratory but also outside of it, in real environments when people make choices and decisions [28]. To decode the information about the brain processing and to understand the data obtained by all these tools, mathematical analyses are needed: the signals' interpretation by the experts taking into account different mechanisms of analysis for each tool and certification of reliability signals. Finally, all of these techniques have strengths and weaknesses: their value is related to the accessibility, the time of analysis, the costs of both the equipment and the personnel time, the possibility to manipulate, and the capacity to be portable as well as affordable [100].

## 5. Brain and No Brain Measure Technologies

For marketing research, starting the analysis from the gaps in traditional measures, it is possible to highlight some advantages and limitations of using these relatively new alternative techniques such as neuroimaging or biosignal analysis, providing a brief analysis on when they are used and what do they measure for each tool. As Reimann et al. [101] confirm, traditional measures, like survey, allow to obtain the subject's judgments after they have finished the task in “*postcondition*” and are based on the ability and willingness of the respondent to accurately report their attitudes or prior behaviours [38]; instead, neuroimaging allows researchers to collect the signal and interpret psychological processes in the brain while people make a task or experience marketing stimuli to highlight a link between consumer behaviour and the neural system.

To do this, when people make a specific task or experience marketing stimuli, the researcher compares the brain activation of the experimental task with the brain activation during a control task. Therefore, neuromarketing research tries to better understand the effects of marketing stimuli on consumers, obtaining objective data using the available technology and advances in neuroscience. But do neuromarketing methods resemble qualitative or quantitative research? Although neuroimaging data collection implies a quantitative approach because it measures brain activity in numbers, neuromarketing research seems to have some aspects in common with the qualitative research. Butler [102] proposes a neuromarketing research model that interconnects marketing researchers, practitioners, and other stakeholders and states that more research needs to be performed in order to establish its academic relevance.

**Figure 3:**
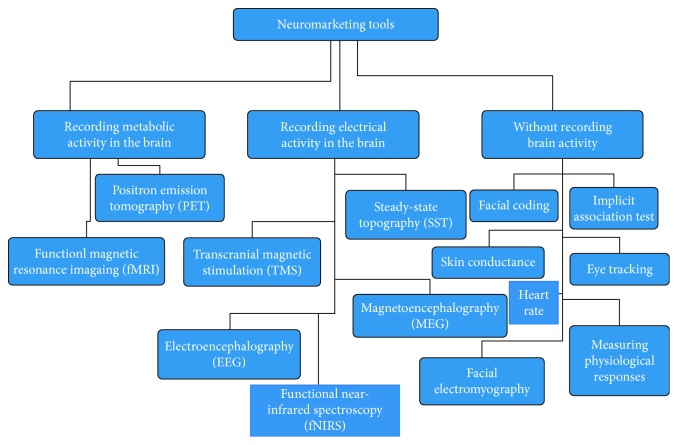
Classification of neuromarketing tools modified from the study of Bercea, 2013.

Zurawicki [103], Kenning and Plassmann [35], and Calvert et al. [104] divide the types of tools used in neuromarketing research into the ones that record metabolic activity, the ones that record electrical activity in the brain [105], and the ones that do not record electrical activity in the brain (Figure 3).

Each of the techniques used in neuromarketing research has specific strengths and weaknesses, which make them more or less appropriate for different research conditions. Another classification can be organized on the basis of the time and space resolutions, and depending on specific neuromarketing studies, certain combinations between techniques seem more appropriate to obtain more information about the marketing issue/research questions. However, we will briefly present each technology based on classification by Bercea and colleagues [100, 105].

### 5.1. Brain-Related Activities: Measurement Technologies

**Figure 4:**
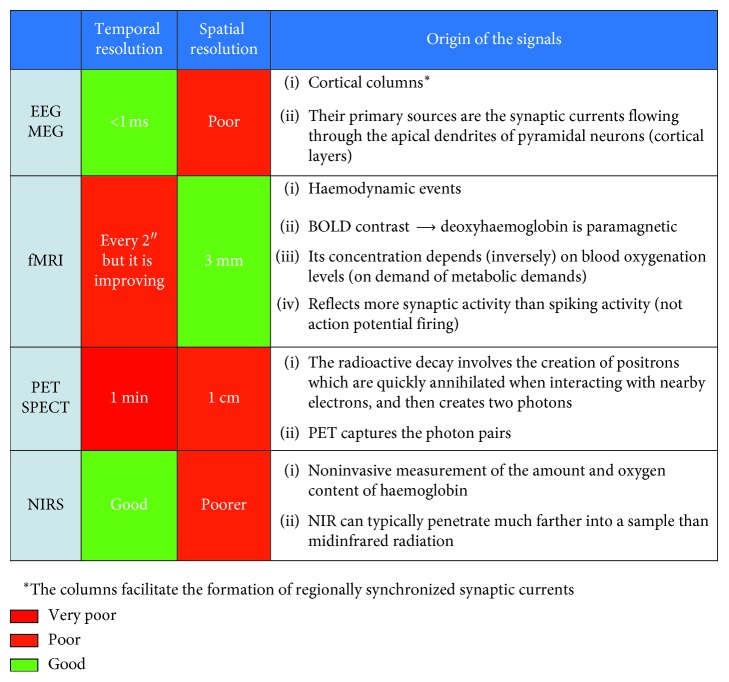
Temporal resolution, spatial resolution, and origin of the signals of the main neuroimaging technique [100].

The fMRI is characterized by high spatial resolution (<1 mm), and its signal is related to metabolic dynamics. Thus, fMRI is a powerful tool for basic research since it is possible to highlight the activations of different brain structures also from deep areas (i.e., behind the brain cortex). In the same application, it is also possible to combine the use of PET or SPECT and fMRI to enhance results with information on what happens at every moment (with PET) and where the change occurs (using fMRI). However, this kind of instrumentation is very bulky, and it requires wide rooms; therefore, it is not possible to use it in realistic conditions, and last but not least, it is very expensive (a machine can cost more than €e100.000). Furthermore, the time resolution is not comparable with that of EEG or MEG (both have a good temporal resolution, <1 ms, but MEG is more expensive to use). The use of transcranial magnetic stimulation (TMS) with EEG or fMRI is a good combination too, as TMS is used in studying causality of specific brain regions for specific mental processes and EEG and fMRI study only correlations between acquired data and stimuli. In general, EEG or MEG can be used as an alternative if the research requires a high temporal resolution for studying, for example, the processing of TV advertisements moment by moment [106]. Functional near-infrared spectroscopy (fNIRS) is a noninvasive optical imaging technique that creates a map of the blood oxygenation in local brain areas during neural activity through examining the cerebral blood flow (CBF) [107]. Brain activation measurement with fNIRS seems to have great potential as it reduces some critical limitations of the fMRI. It is mobile and lower in cost, enabling use in real-world situations for freely moving subjects [108–111]. As it is comfortable and tolerant of body movements, it is highly portable, and it is described as a major innovation in neuroeconomic research [112]. Despite the novelty of fNIRS in neuroscience, the reliability and validity of the method to measure cortical activation have been shown in a wide spectrum of studies inside and outside the laboratory. Studies trying to focus on more realistic and natural environments have used fNIRS, for example, while walking in a city [112], driving a car [113], flying an airplane simulator [7], playing table tennis and piano [114], or focusing on a realistic grocery shopping atmosphere [115, 116]. In Figure 4, the main differences among different neuroscientific tools, in terms of temporal and spatial resolution, can be seen.

Considering their pro and cons, it can be derived that the joint use of some of these techniques with the traditional marketing research methods (as neuromarketing alone is not always capable of answering the research questions) could lead to better results, capable of finding new valuable consumer insights and revolutionizing marketing research itself. According to the research questions and objectives, in fact, there is a proper neuromarketing technique. The tools presented are the key points of understanding mechanisms underlying consumer behaviour, and they add value to traditional marketing research techniques. Using them, researchers can discover what people do not what to reveal and what exactly influences their decisions, even things they are not aware of [105].

### 5.2. No Brain-Related Activities: Measurement Technologies

#### 5.2.1. Heart Rate and Galvanic Skin Response

Measuring emotion is one of the most widespread aims of many scientists' research studies. There are a few dimensions that organize emotional response. The two most common ones are valence and arousal. The first one contrasts states of pleasure (e.g., happiness) with states of displeasure (e.g., sadness), and the second one contrasts states of low arousal (e.g., quietness) with states of high arousal (e.g., surprised) [117, 118]. Specifically, the galvanic skin response (GSR) is typically quantified in terms of the skin conductance level (SCL) or short-duration skin conductance responses (SCRs), while the most commonly used cardiovascular measure is the heart rate (HR). Using devices able to record the variation of the GSR and HR, it is possible to monitor autonomic activity and to assess the internal emotional state of the subjects. In fact, the GSR is considered a sensitive and convenient measure for indexing changes in sympathetic arousal associated with emotion, cognition, and attention [119]. Instead, several papers reported that the HR correlates with the emotional valence of a stimulus, e.g., the positive or negative component of the emotion [120]. Moreover, in experimental psychology, the circumplex plane of affects has been proposed and used, in which emotions are mapped in a two-dimensional space where horizontal and vertical axes are related to valence and arousal, respectively [118, 121]. Thus, the joint measurement of the HR and GSR and their positioning on the affect circumplex return the emotion perceived by the subject during a specific experimental task [61]. Even the HR and GSR could be used simultaneously with other tools (i.e., EEG) to obtain information about both the emotional and cognitive responses.

#### 5.2.2. Eye Tracker

To get information about where people look and to track their eye movements, the eye tracker (ET) tool has a special place among modern neurophysiological techniques. It allows to measure different processes of the human brain to salience stimuli, giving a useful insight into advertising and marketing stimuli. Based on the relationship between visual attention and eye movements [122], the ET is an effective tool for experimental psychology and neurological research. It detects eye position, gaze direction, a sequence of eye movements, and visual adaptation during cognitive activities and allows users to analyse behaviour and cognition by exploring the subject's gaze. It records where and what the person is looking at (fixations), the time of fixations spent on a specific area of interest (AoI) on the stimulus, the movement of the eyes in relation to the subject' head to get information about specific patterns of visualization, pupil dilation, and the number of blinks [103, 123, 124]. The main types of eye movements which can be detected by ET are saccades, smooth pursuit eye movement (SPEM), and vestibuloocular reflex [125]. Eye fixations usually range from approximately 200 ms during the reading of a text to 350 ms during viewing of a scene. The saccadic movement to the new target takes approximately 200 ms [126]. The resulting series of fixations and saccades is called the scan path and allows to analyse visual perception, cognitive intent, interest, and relevance [103]. There are different techniques for measuring the movement of the eyes: contact lens-based, electrooculogram-based, and video-based [127] eye trackers. The latter is the most commonly used that captures the gaze while the viewer looks at stimuli and provides a more comfortable alternative to the electrooculography-based ET. More performing devices facilitate obtaining a three-dimensional representation and capturing fast movements of the eyes, such as microsaccades. They provide a quantitative and qualitative analysis of the gaze, which is very useful in understanding the choice and perceptual decision-making. In the high-tech era, the ET has several applications related to the interaction between humans and computers. In marketing studies, the ET is usually used to evaluate the customer' reactions to information on websites, different packaging product designs, the spatial orientation of attention, the performance in visual tasks, the customer response to the advertisement or to the shelf in a store, and the emotional and cognitive impacts of various spurs on the brain [75, 128]. Additionally, the ET can be used in both laboratory settings and field environments [129].

Understanding the mechanisms that guide consumers to select specific elements in an image has many applications in the business world [130]. Therefore, the ET can make available information on what is more appropriate to the involvement of attention, as it is related to patterns of visual fixations, in many different marketing issues [131]. The ET can also be used with other tools (i.e., EEG) to measure cognitive and emotional responses and lead synergy for new insights, particularly in relation to consumer behaviour and marketing communications.

Other eye-tracking uses have been reported by Chaen and Lee [132] in several studies such as usability, marketing, cognitive psychology, and behavioural psychology. Thanks to eye-tracking results, more effective ways of producing online sales and difficulties during the customer checkout process are identified.

Orquìn and Loose [133] argue that the eyes movements during the decision-making process depend on the requirements of a given task and on the characteristic of the stimuli (that are causing a bias to capture information) where salient visual stimuli are preferred. Finally, it is possible to distinguish two factors that contribute to attention and influence the meaning of a stimulus to an individual, and they are top-down and bottom-up factors [134]. To Pieters and Wedel [135], bottom-up factors are intended as the characteristics of the stimulus itself, and they are a rapid form of attentional capture. Instead, the second one is previous ideas about the product that a consumer already had. Top-down factors require consumers to voluntarily search and pay attention to specific information [136].

#### 5.2.3. Facial Expression

Human face is considered to be the richest source of information among nonverbal channels for emotion expression [137]. In everyday life conditions, humans frequently exchange social information since they are a highly social species, and one of the richest and most powerful tools in what is called “*social communication*” is the face, from which people can quickly and easily get information about identity, gender, sex, age, race, ethnicity, sexual orientation, physical health, attractiveness, emotional state, personality traits, pain or physical pleasure, deception, and even social status [138]. The analysis of facial expression in market research studies is very useful, and one of them has been conducted by Hazlett and Hazlett [139] who revealed that facial electromyography (fEMG) can return information about the perception of different commercials. Somervuori and Ravaja [140] reported that when people look at a static image, the activity of the zygomaticus major, a muscle responsible for smiling, may serve as a good predictor of purchase decisions. Another study [141] used a specific software program to record and analyse facial expressions to construct online purchase decision classifiers. Through these classifiers, it was possible to predict the online purchase substantially above the chance level. Also, another model based on the activity of facial muscles was able to forecast purchase intentions with 78% accuracy [142], and from these results, it seems that the predictive power of facial expressions remains regardless of the software used to gather and analyse the data. For instance, Lewinski et al. [143] used in their study FaceReader 5.0, provided by Noldus: also in this case, the usefulness of facial expression analysis in assessing the effectiveness of marketing video messages is confirmed [144]. In a similar way, FaceReader was applied to measure the intensity of human emotions in the static advertising context [145]. Notably, FaceReader enables researchers to identify real-time facial expressions of customers and classify emotions into diverse types [146]. FaceReader can combine both basic emotions and dimensional emotion approach (i.e., arousal and valence) [146].

#### 5.2.4. Reaction Time Test

To measure brand perception, brand awareness, degree of positivity, or negativity associated with a product, image, or brand, it is possible to use indirect measures based on the testers' reaction time to a stimulus during a comparison process among two or more stimuli. In order to overcome the lack of inner knowledge on consumer behaviour based so far only on questionnaires, consumer psychologists have developed these techniques that rely on nondeclarative features of people's responses [147]. Those indirect measures are used to predict consumer behaviour. This approach measures consumers' reaction times and/or accuracy on tasks that are systematically biased by their reactions to brands or ads and measures the buyer's attitude. Implicit associations are linked to unconscious automatic attitudes. There is evidence that brands have implicitly engaged in specific positive associations (e.g., quality, value, youth, strength, and speed). Such implicit associations may be critical for the consumer's decision to buy products/services [148]. There are different examples of such methods based on reaction time (RT), for instance, the implicit association test (IAT [149]), the extrinsic affective Simon task (EAST [150]), and the implicit relational assessment procedure (IRAP [151]) as well as various lexical decision tasks where primes and targets are sequentially presented [152, 153]. In all these mentioned cases, the aim is to identify the presence and quantify the strength of semantic or evaluative associations between objects and attributes.

Implicit association methods have a long history, but the IAT was introduced in the scientific literature in 1998 by Anthony Greenwald et al. [149] to measure individual differences in implicit cognitions because at the beginning, the IAT was used to study racial attitudes, self-concept, and self-esteem. Currently, the IAT is used in different areas, including consumer attitudes (toward brands or categories), and it can be considered one of the neuromarketing tools. Specifically, the IAT measures the underlying attitudes (evaluations) of the subjects by assessing reaction times on two cognitive tasks, identifying the speed with which they can associate two different concepts (stimuli such as advertisements, brands, and concepts) with two different evaluative anchors (attributes). As Zurawicki [103] states, measuring the reaction time between stimulus appearance and its response (response time or reaction time) can inform researchers about the complexity of the stimulus to an individual and how the subject relates to it. A shorter latency time in recognition or association (of positive or negative adjectives) to a stimulus indicates a more embedded attitude towards that particular stimulus. Although the difference in response is a few milliseconds, this is a very effective indicator for the assessment of the preference. This method can be used on recalling studies or on measuring the subject's attitude towards certain stimuli [105].

In Table 1, a list of the main neuromarketing tools can be seen, in addition to what they measure, when they could be used, and their advantages and limitations.

**Figure 5:**
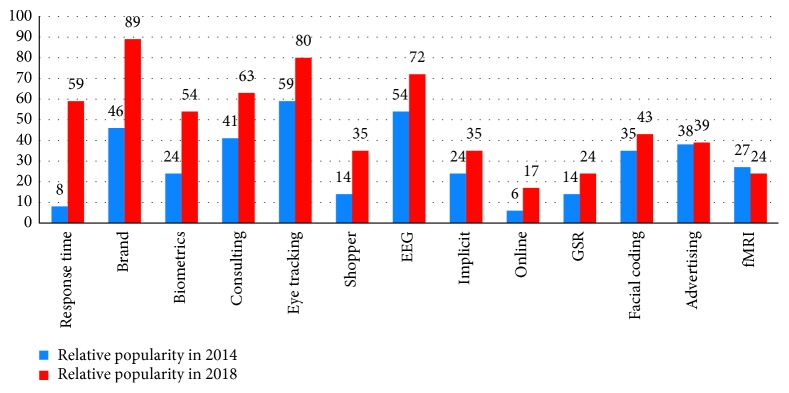
Relative popularity of selected methods in 2014 vs. 2018. Source: https://bit.ly/2UbeWzz.

In a survey conducted by the NMSBA (https://bit.ly/2UbeWzz), the results show that the mix of methods and tools offered by neuromarketing vendors in 2018 is quite heterogeneous and quite different from the mix that existed as recently as four years ago. Through a survey, the respondents were asked which methodologies, kind of services, techniques, and equipment they used in their marketing research studies. The NMSBA ranked methods by their relative popularity compared to the most-often mentioned specialty offered in 2014 and in 2018 by neuromarketing vendors listed in NMSBA company directories. In Figure 5, all the relative popularity of selected methods is shown.

As can be seen from the figure, the fMRI shows a small decline during the period 2014–2018; instead, the response time studies have had the greatest increment in popularity in the same period. Brand measurement also shows a growing trend: this probably happens because response time techniques opened new possibilities for exploring the implicit mental connections that consumers make with the brands. Similarly, in 2018, vendors looked much more for services like biometrics, consulting, eye tracking, and shopper studies than they were in 2014. The neurophysiological techniques, from the most to the least offered by vendors, were EEG, eye tracking, various biometrics, implicit response tests, surveys, facial coding, and interviews. In contrast to the increased focus on brands, references to advertising research have remained relatively flat.

## 6. Main Brain Areas and Processes of Interest for Consumer Neuroscience

The main aim of neuroimaging technique application in consumer neuroscience is to measure processes such as decision-making, reward processing, memory, attention, approach and withdrawal motivation, and emotional processing, by means of specific brain area activations. Advanced neuroimaging techniques facilitate precise identification of neural responses across specific brain regions. This anatomical localization is important for consumer research because of the role specific brain regions might play in different cognitive and emotional functions [30]. The following section will describe the main brain regions and processes and some (most popular) indexes cited in scientific publications.

### 6.1. Decision-Making

One of the leading questions in marketing research focuses on consumers' decision-making processes: how does a consumer cope with different product alternatives based on personal perceived benefits and costs? Several regions of the prefrontal cortex (PFC), situated in the frontal lobe of the brain, play an important role in the underlying processes of human decision-making. Several studies in particular highlight that both the orbitofrontal cortex (OFC) and the ventromedial prefrontal cortex (VMPFC) are involved in decision-making processes by assessing the (perceived) value of different options and potential outcomes [154, 155]. Importantly, the OFC is associated with the evaluation of trade-off and the expected capacity of the outcomes in terms of the capability to satisfy one's needs [156]. It plays a central role in choosing appropriate behaviours, especially in unpredictable situations [157]. The dorsolateral prefrontal cortex (DLPFC) plays a critical role in decision-making as well, given its involvement in cognitive control over emotion [158]. In particular, it contributes to impulse control for complying with social norms, while the ventrolateral prefrontal cortex (VLPFC) potentially plays a role in motivating social norm compliance by projecting the threat of punishment from others in case of noncompliance [159]. The cognitive effort in the PFC interestingly appears to be higher in risky situations as compared to when a sure gain is expected [160]. Measuring the activity of these regions can thus provide useful insights into marketing constructs such as perceived value and the neural foundations of consumer choices.

### 6.2. Reward Processing

Other brain regions which respond to subjectively attractive rewards such as food [161], money [162], and drugs [163] are involved in the reward system process. Attractive elements like product design or a preferred brand can be considered rewarding stimuli within consumers' brains, which may trigger the psychological motivations that influence purchase behaviour. To monitor the reward process activated by stimuli, the activation of the striatum—a striped mass of white and grey matter located in the basal ganglia inside the forebrain—can be measured. While planning and controlling movements are the main function of the striatum, it also plays a role in the brain's reward system; there is evidence of the role the striatum and its components (putamen, caudate nucleus, and nucleus accumbens) play in the evaluation of one's expectations compared to actual rewards received [164] and the influence of social factors on this region's reward-related activity [165]. Even the ventral tegmental area (VTA) is part of the reward system, which passes the neurotransmitter dopamine to other brain regions, enabling the modulation of decision-making and affecting in goal-seeking behaviours [166].

### 6.3. Attention and Memory

Every day, consumers are exposed to an enormous amount of information, even though their processing capacity is limited. Each second, people receive 11 million bits of unfiltered information (in the form of advertisements, products, brands, images, colours, sounds, etc.) through all their senses. Most of the input goes by unnoticed, given that humans are capable of processing a small part of it, around 50 bits [167]. It is reasonable to assume that it has a profound influence on consumer behaviour of how they represent, attend to, and perceive incoming information. A key question is what consumers direct their attention towards (i.e., focus on) once they are exposed to several rapidly identified choice alternatives (i.e., brands and communications). Paying attention to something, a mechanism of selecting information that gets prioritised over other available information, means a person needs to be aware they are paying attention to it. A recent neuroscientific review identifies four conceptual components, fundamental to attention: bottom-up or saliency filters, top-down control, competitive visual selection, and working memory [168]. Since aesthetic components are a prominent part of advertisements, logos, and product designs, they are components processed by the brain in the form of visual stimuli and are deconstructed by the brain into their constituent elements such as shapes and colours [30]. For this reason, the underlying brain mechanisms of attention and visual processing are for sure of interest for consumer research. The prefrontal cortex directs towards and focuses on attention and has been shown to connect with the neurons responsible for processing visual stimuli in the occipital lobe, the vision centre of the brain [169].

Similarly, studying memory-related mental processes might provide useful insights into variables influencing consumer behaviour such as brand awareness, product experience, and advertising recall. Within the very complex memory variable, marketers are above all interested in encoding or storing memories in retrieving or remembering memory processes as well as in short- and long-term memory processes [170]. Furthermore, to perform complex cognitive tasks, people must maintain access to large amounts of information. The hippocampus, located in the temporal lobe, plays a major role in generating different forms of memory as well as in memory processing and consolidation [171], i.e., in long-term memory, and in the acquisition and recall of declarative memory [172]. Additionally, the amygdala, situated next and closely related to the hippocampus, is an important modulator of the memory system, particularly in memory consolidation [171].

### 6.4. Mental Workload

While making decisions, people invest effort to process information, coming from both the external environment and personal knowledge. In cognitive psychology, this process is called mental workload, and its theory was developed out of the study of problem-solving by John Sweller in the late 1980s [173]. A unique definition of it doesn't exist, but specifically for the operative environment, it has been given various definitions in the last decades. For example, Gopher and Donchin (1986) defined mental workload as a hypothetical construct that describes the extent to which the cognitive resources are required to perform a task actively engaged by the operator [174]; Hart and Staveland (1988) considered workload as the process that emerges from the interaction between the requirements of a task and the circumstances under which it is performed and the skills, behaviours, and perceptions of the operator [175]; Eggemeier and Wilson (1991) defined mental workload as “*the amount of the resources required to meet system demands*” [176]. However, mental workload cannot be considered a unitary concept because it is the result of different aspects interacting with each other. Psychophysiological measurements are often used to evaluate the level of cognitive demand induced by a task [21].

Characteristic changes in the EEG spectra reflecting levels of mental workload have been identified in different works [25, 177–179]. Several studies described the correlation of spectral power of the EEG with the complexity of the task that the subject is performing. In fact, an increase in the theta band spectral power (4–7 Hz), especially on the frontal cortex, and a decrease in the alpha band (8–12 Hz), over the parietal cortex, have been observed when the required mental workload increases [23]. Because of its close relationship with human performance (i.e., the human performance generally decreases when mental workload becomes too high or too low), it is considered a very relevant mental concept in cognitive neuroscience applied to those fields where human decision-making is crucial, such as neuroeconomics and neuromarketing. In marketing research, it is very important to measure this index when customers are involved in a specific operational task. It could be useful, for instance, in a website usability test, when people look for a landing page, or when people visit a store, or in general for whatever cognitive task relevant in a marketing research.

### 6.5. Approach and Withdrawal Motivation

When people interact with a stimulus (i.e., a product, a brand, an image, etc.), they can be either attracted to it or not. Contemporarily, researchers attempt to investigate brain activity signals correlated with an increase of emotional involvement during the interaction with marketing stimuli [180, 181]. Researchers have found strong relationships between people's behavioural approach system (BAS) and behavioural inhibition system (BIS) and their consumer-related activities. Indirect variables of emotional processing in fact could be gathered by tracking the activity of specific anatomical structures' variations linked to the emotional processing activity in human beings, such as the prefrontal and frontal cortexes (PFC and FC, respectively) [182]. In particular, the structurally and functionally heterogeneous PFC region plays a well-recognised role in the generation of emotions [183]. EEG spectral power analysis indicates that the anterior cerebral hemispheres are differentially lateralized for approach and withdrawal motivational tendencies and emotions. Specifically, findings suggest that the left PFC is an important area in a widespread circuit mediating appetitive approach behaviour, while the right PFC appears to form a major component of a neural circuit instantiating defensive withdrawal [183, 184]. fMRI studies have shown that, at the time a reward is being enjoyed, activity in the orbitofrontal cortex (OFC), in particular in its medial parts, correlates with subjective reports about the pleasantness or valence of the experience. An interesting open question is which neural systems encode negative experiences. Several studies imply that unpleasantness of taste correlates with brain activity in the lateral OFC and left dorsal anterior insula/operculum [185, 186]. O'Doherty and colleagues found that the size of abstract punishments (i.e., losing money) activates lateral parts of the OFC [187]. A problem in investigating negative experience is to dissociate it from intensity, given intensity's negativity bias: negative experiences are usually perceived to be more intense and thus are often confounded [186], in particular for visual stimuli such as facial or object attractiveness. As previously presented, by utilising a different methodological approach to investigate positive versus negative emotional experiences, neuromarketing studies are based on the idea that there is a left-right asymmetry of frontal EEG signals [188]. Related studies suggest that relatively greater activity in the left frontal region is associated with either positive emotional experience or the motivational drive to approach an object [30]. The approach-withdrawal index [188] is considered in several neuromarketing studies to evaluate TV commercial advertising, to investigate the consumer's gender differences during the observation of TV ads, and to evaluate the olfactory stimuli in young subjects [90], in user experience research studies [189], in the context of online interactive shopping environments [190, 191], and during neuroaesthetic studies [192–196] and taste experience [197–199].

### 6.6. Emotional Processing

Emotions drive consumer choices and are very important in the decision-making process. The amygdala, a central brain structure, has a pivotal role in regulation of emotional responses. It is involved in the processing of negative emotions and unknown stimuli, as well as in aversive responses to inequity [159]. It is also known as a locus of aversive and fear memory. Concerning positive emotions, it has been shown to be involved even if with a minor extent than for negative emotions, usually in relation to rewarding stimuli [200]. Another key emotion-related region is the insula (or insular cortex) which plays an important role in the processing of negative experiences such as the perception and expectation of risks, especially when making decisions for which a social or financial risk is expected [201, 202]. Likewise for the amygdala, the activation of the insula has been associated with anger and disgust in response to unfair economic situations [179]. Additionally, as well as being involved in the evaluation process of stimuli, the aforementioned OFC plays a role in experiencing and anticipating the emotion of regret when outcomes differ from expectations [203]. Finally, another area that evaluates emotional and motivational information is the cingulate cortex, which includes the cingulate gyrus. It integrates the emotional information in the decision-making process [30, 204]. Moreover, the anterior cingulate has been associated with the experience of an internal conflict between alternative options, and its activation could reflect the conflict between cognitive and emotional motivations [179]. The role of emotions in decision-making has been further explained through neurological and cognitive frameworks such as the somatic marker theory [205]. Overall, the study of these brain mechanisms is likely to be central in consumer neuroscience because of the importance of the emotional component of purchase decisions in traditional consumer research [206]. However, there is not a single brain region responsible for emotional processes, and no single brain region is activated in relation to one particular type of emotion as the interconnected cerebral network involved in emotion is really complex [30, 207]. Nowadays, it is also possible to assess the emotional state of the subject by monitoring autonomic activity such as the heart rate (HR) and skin conductance (SC). Indeed, emotions are accompanied by (bodily) reactions that are partially beyond an individual's control. These autonomic reactions include facial expressions (e.g., smiling and frowning) and physiological reactions (e.g., sweating) primarily caused by changes in the autonomic nervous system (ANS) [208, 209]. In fact, the autonomic reactions are manifestations of lower-order emotional processes. Over the years, as the validity of self-reports for measuring lower-order emotions is often biased by cognitive or social desirability constraints, several instruments have been developed to capture autonomic reactions. So, the measurement of autonomic reactions can overcome the validity problem of self-report because they measure emotional responses beyond the respondents' control. SC or electrodermal activity is a frequently used measure of activation of the autonomic nervous system [210]. SC gives an indication of the electrical conductance of the skin related to the level of sweat in the eccrine sweat glands. These sweat glands are involved in emotion-evoked sweating. They cover the whole body but are most dense on the palms and the soles of the feet [210]. When there is more activation of the autonomic nervous system, there will be more sweat secretion and consequently a higher level of SC. Because the increase in activation of the ANS is an indicator of arousal, SC can be used as a measure of arousal [211]. When measuring SC in real settings, electrodes that register the level of conductance (or inversely, resistance) to a light electrical current are placed on the sweat-sensible places in the palm of the hand.

Regarding the HR, in psychophysiological research, it is mostly operationalized as the number of milliseconds since the previous heart beat [212]. To distinguish HR measures indicating attention to commercials from measures indicating arousal responses to commercials, Lang [213] looked at phasic (i.e., short term) changes in the heart rate for attention and at tonic (i.e., long term) changes as an indication of arousal. She concluded that, for both attention and arousal, the heart rate can be a valid, real-time, and continuous measure. When attention increases, there is a phasic deceleration in the HR. Arousal, furthermore, is accompanied by a tonic acceleration in the heart rate. At the same time, the heart rate can give an indication of the valence of an emotional response. Compared to neutral stimuli, both positive and negative stimuli first exhibit a phasic decrease in the HR. At a tonic level, positive stimuli evoke an increase in the heart rate, while negative stimuli generally lead to a decrease in the heart rate [214, 215]. These findings were replicated in advertising studies using the heart rate to measure emotional responses to advertising stimuli [213, 216]. The HR is mostly not detected directly in the heart but in other—more convenient—places such as the fingertips. Placing a device that registers the heart rate on one finger has the advantage of requiring little interference with the subject (noninvasiveness). In this way, the registration of the heart rate can be considered an easy and cheap way to measure psychophysiological reactions evoked by advertising and other marketing stimuli [212].

## 7. Value Proposition and Marketing Operation: How Neuromarketing Can Improve Customer Value?

The neuromarketing applications have grown significantly in the last years in each marketing area such as communication, product, packaging, brand, retail, and pricing. The following section will describe, for each area, the main studies conducted so far and in which way neuromarketing tools can improve the customer value.

### 7.1. TV Commercial Advertising and Public Service Announcement (PSA)

“*I know that half of money spent in advertising is wasted, but I don't know which such half is*”: these are the words that John Wanamaker, the builder of the first mall in the US in 1876, said joking [217]. Every year, companies spend huge amounts of their budget on advertising campaigns, with growing questions among managers as to how effective and profitable these expenses might be.

Advertising is the marketing area that has benefited the most from neuromarketing techniques. If it is true that the average of consumers' purchases is made with the heart and justified with the mind, it is necessary to create campaigns that stimulate both hemispheres, right more emotional and left more rational. Every day, people are exposed to several stimuli, and they watch or listen to even hundreds of ads that are placed in mass media (TV, radio, Internet, press, etc.) but are also visible in other places (streets, buses, mailboxes, stadiums, etc.) [218]. The question of how the brain processes and stores advertising stimuli may be of essential importance [219]. In the age of multimedia and multitasking, finding ways to get consumers' attention, emotion, and memorization has become a primary focus of the advertisers. Providing entertainment in ads is regarded as an effective approach to capture the consumers' initial attention and interest in viewing the entire ad [220] and to enhance the message memorization; however, it has not always been like this. Many years ago, in the fifties or sixties, the most television ads demonstrated product features and concentrated on selling [221].

Today, some ads are probably more humorous than their programs, and millions of viewers choose to watch commercials online for their entertainment value on social networking sites such as Facebook or YouTube. Since television is mainly used as an entertainment medium, it is not surprising that advertising is well perceived in this medium as entertaining and creative. For example, entertaining content has been shown to increase brand purchase intentions by reducing the consumer's resistance to persuasion [222]. Thus, the measurements of the emotional correlates of the observation of TV ads could give additional information besides that already obtainable with the traditional methodologies (e.g., questionnaires and verbal interviews).

For the analysis of the impact of a TV commercial, nowadays it is possible to obtain, through consumer neuroscience models, information about the following:

- Evaluation of efficacy of an advertisement by neuromarketing indexes as a whole or for particular frame segments (i.e., introduction, product, service, testimonial, claim, brand, payoff, etc.)
- Measurement of different impacts on the perception of the TV commercial by two or more subgroups (male, female, young, adult, user, no user, etc.)
- Definition of a reduction criteria of TV commercials in time, based on neuroindicators and producing a shorter but also effective version (i.e., 30″ to 20″ or 15″)
- Pretesting of an advertisement
- Analysis of the impact of repeated exposure (to test the optimal grossing rating point (GRP))
- Measurement of what and where people look at on a screen, when attention is placed on certain advertising visual elements and how long each fixation lasts for

The impact of advertising on the recipient depends on several different factors, such as the type of the product, the nature of the target group, or the value of the decision for the consumer [223]. However, the effectiveness of achieving the intended goals at some steps can be well analysed by applying modern research techniques, such as cognitive neuroscience techniques. It is possible to examine which emotions are caused by particular scenes of advertising, which elements of advertising the recipients were paying particular attention to, or which was remembered [218, 224, 225]. The main point is to link—in terms of superior cognitive functions—a particular neuroelectrical brain imaging state to an index related to the “*success*” of the TV ad analysed rather than to the content of the brain. However, it is not easy to know a good metric for the success of a TV advertisement. In fact, the historic trend of the selling of the product related to the advertisement broadcast and the cost of an advertisement or advertising program are known by the company placing the advertisement, and the value, the GRP, and the effectiveness of ads performed are less apparent and usually unknown to the scientific researchers.

From an economic and marketing perspective, the aim of a neuroscientific approach is to get a better understanding on how mass consumer advertising of (established) brands affects the brand systems themselves. From a neuroscience perspective, the broad goal is better understanding of both the neural mechanisms underlying the impact of affect and cognition on memory and the neural correlates of choice and decision-making [44]. Several studies have been conducted to evaluate the efficacy of commercial advertising.

Ioannides et al. [226] have employed MEG to study the neuronal responses in subjects viewing the same TV advertisements as used by Ambler and Burne [227]. The results show that cortical centres associated with the executive control of working memory and maintenance of higher-order representations of a complex visual material are activated by cognitive advertisements rather than affective ones. Interestingly, neuronal responses to an affective visual material seem to exhibit a greater intersubject variability than responses to a cognitive material. Young [228] has used the EEG to detect putative “*branding moments*” within TV commercials. Other neuromarketing studies have been conducted for the assessment of the efficacy of TV advertising stimuli [44, 61, 181, 229–235], to investigate the consumer's gender differences during the observation of TV commercials [236, 237].

Typically, these are commercials, but there are other advertising categories, such as social issue and political advertisements. Social campaigns, as well as commercials, are based on similar principles and techniques, although their goals are different. The aim of commercial advertising refers usually to advertising products or services whose sale will bring measurable benefits and profits. On the contrary, the aim of the social campaign refers to providing and widening of social knowledge, engaging in social affairs, or sensitizing to certain issues [238]. Nonprofit organizations (NPOs) rely on donations to keep functioning and to continue making an impact, and each detail needs to be carefully thought in order to promote their cause and to encourage people to donate and support them, or to sensitizing to certain behaviour. The rules determining the effectiveness of social issue advertising are comparable to the rules of commercials' assessment. Advertising is effective when the recipient notices and then remembers a content which are the intent of the message (company logo, product name, name of a candidate in election, desired social behaviour, call to action, etc.). If the result is different, it means that an ad is pointless. A vast majority of social issue advertising is based on emotions, and these are usually messages associated with fear or compassion. NPOs are often told that, to attract funding, they need an emotional appeal, but which kind of emotional appeal? Fear is usually used in social issue campaigns, which focus on care of oneself or one's family welfare (to quit smoking, to drive safely, etc.), while compassion is where one should help others (giving blood, helping the hungry people, etc.) [239]. However, heavy guilt-tripping messages could produce an ineffective reaction when using overly negative images, or at the same time and analogously, the arousing of only positive emotions can have an unsuccessful effect. Methods of public service announcement (PSA) evaluation are often performed *a posteriori*, while an appropriate pretesting of the PSA material would be extremely useful to check the impact of the particular creative solutions on the target populations. It could be of interest to understand if the PSA assessment (e.g., effective or ineffective) can be performed through the study of the neurophysiological reaction to the exposure to the PSA itself. It could be hypothesized that possible different cerebral patterns could be obtained in response to different kinds of effective (e.g., successful) or ineffective PSAs or in the perception of the consumer's gender differences during the observation of charity campaigns by using neurophysiological measurements, such as EEG [240]. Therefore, obtaining measurable neurophysiological parameters, collected through direct analysis of the measured cerebral/emotional/visual attention (VA) in response to the observation of PSAs, represents an important question [73]. PSAs are at the core of many public health campaigns against smoking, junk foods, abuse of alcohol, and other possible threats to the health of citizens. But the content of these PSAs could also be directed for the promotions of “*positive*” social collective behaviour, for instance, calling against racism, supporting the integration of different cultures in the country, or promoting a healthy drive style, for the road security. Therefore, effective PSAs provide a great public health benefit [241, 242]. Using neuromarketing tools, in the last years, several studies have been conducted to develop more effective social campaigns, such as promoting the encouragement of the use of seat belts in cars or promoting smoking cessation with antismoking campaigns [52, 70–75].

### 7.2. Product Choice

It is easy for a company to keep track of what people buy, but it is harder to figure out why. Despite the laborious process to design and select the products, a large portion of them turns out to be a failure because they do not meet the customers' expectations or needs. These unpopular designs generate large amounts of unsold stocks, which end up being sold at discount prices. Several fundamental marketing factors—such as inadequate pricing, design and packaging, or the position of the product on the supermarket—may cause these failures. As a result, (a) the satisfaction of customers decreases as they cannot find their desired products in stores, (b) the brand image is devalued [243], and (c) customers lose faith in the brand and tend not to come back to the shop but instead shift to another vendor or get used to buy only at discount prices. These damages are long term and hard to recover as in competitive commercial markets, regaining customer trust and rebuilding brand image are expensive tasks which may take years. So, it is very important to consider these aspects during the product marketing strategy, and the main questions of many industries are if there is a way to reduce the chance of failure and if it is possible to develop a predictive tool that could predict the success of a product even before it is launched, very finely tuned to customer expectations and desires [244].

As far as it concerns the product choice, the consumer's choice is like a complex sequence of cerebral activations. From a behavioural point of view, choices with a high probability are faster than those less predictable. This can be interpreted by supposing that, in the case of more choices, all difficult, the cortical activities are more complex than the activity that would occur in the choices simple to make.

Hence, as the ability of neuroimaging to predict or influence postdesign purchase decisions seems to be limited, neuroimaging may be better suited to gauging responses before products are launched on the market. The primary reason is that neuroimaging may yield insights into the product experience itself, allowing to compare different premarket products proving information about which is the best to put on the market.

In fact, the neuroscience contribution to the world of management highlighted that the rational component of decisions counts little: on a scale from 1 to 100, it counts 5%. This is one of the reasons why up to 80% of new product launches fail within a year [245]. For decisions, the irrational component is more important, and neuroscience is providing a great help to marketers to better understand how and why consumers choose and what are the levels of cognitive and emotional involvement activated during the perception of a product during the decision-making process.

People are generally not able to reconstruct and interpret their thoughts and feelings, which is why self-reports often do not yield the desired information about consumers' real opinion of a product. For example, self-reports are frequently in contrast with the actual inner states of the subjects [208]. In product choice, consumer neuroscience can yield a more complete and objective understanding of a consumer's inner desires and may consequently assist companies to fine tune their strategies according to the latter. One important aspect of product policy is the optimal design of a product according to the preferences of the customer [246].

For example, the investigations by Erk et al. [247] provided the first central insights into how the brain processes differently designed goods (e.g., sports cars, limousines, and small cars). The study conducted with the fMRI showed that reward-related brain areas are activated by objects that have gained a reputation as status symbols through cultural conditioning that signal wealth and social dominance: picture of some cars, in fact, led to activation of the left anterior cingulate cortex, the left orbitofrontal cortex, and the bilateral prefrontal cortex, as well as the right ventral striatum. According to the present standard of knowledge, these regions are associated with motivation, encoding of rewarding stimuli, prediction of rewards, and decision-making [248, 249]. A very interesting finding for the optimal design of a product is that the authors reasoned that the relative activation in the ventral striatum, in which the nucleus accumbens is located, can be seen as an indicator for how attractive a visual stimulus (i.e., product design or shape) is evaluated to be [219].

### 7.3. Packaging

The role of packaging in marketing is becoming increasingly important, as it is one of the main product attributes used by companies to distinguish their products from competitors. In order to be competitive among many others, the company must study how to make packaging attractive and immediately recognizable among thousands of products in a store. The visual aspect of packaging is a very important carrier of a specifically encoded market communication system. It is a particular form of language that should lead to attracting the consumer's attention to a product and then decode the message, generate interest, trigger a purchase decision, and leave a long-lasting positive connotation [250]. The role and importance of packaging are demonstrated in many studies for its ability to communicate relevant product information, its influence on consumers' attention, perception, and purchase intentions [251, 252]. Expressions such as “*the silent salesman*” [253] are commonly used to describe the role of packaging. Packaging has become a significant marketing channel because of its presence in the shops, combined with its strong influence on customers' decisions [254]. More recently, there has been a growing interest surrounding the influence of the sensory characteristics of packaging on consumers' expectations and on consumers' subsequent food experience [255, 256]. In fact, the product could be perceived as a combination of different items: the package, the brand, the aesthetic side (colour, graphic, image, and shape), and the context of usage [257]. Each of these items may elicit different cognitive and emotional reactions with different meanings for consumers. Several studies have demonstrated that packaging has an important role both in the moment of the purchase and during the phase of usage and usability of the product, known as the first and the second moment of truth, respectively [258, 259]. To be considered useful and effective, the package must be easy to use; the information on it must be relevant so that consumers do not misuse the product; it must fit in storage spaces; if the product should be dosed, the package must facilitate this; and so on. In addition, when it refers to services, companies should design packages with user-friendly prerequisites because there are no employees present during the service consumption process. In such a case, the package could be considered the component that bridges the gap between production and consumption [260].

In 2005, Campbell was one of the first companies to decide to use neuromarketing techniques to know which factors lead consumers to choose on buying a soup, and if a new label design would enhance the sales of the soup. Campbell spent two years studying the emotional and cognitive reaction in response to pictures of bowls of soup in logo design. Throughout the results obtained, Campbell redesigned its labels [261].

In one fMRI experiment, Stoll and colleagues [262] measured the brain activity of subjects who had to make decisions about the attractiveness of certain fast-moving consumer good packages finding that attractive and unattractive packages can trigger different cortical activity changes. The study revealed significant cortical activity changes in visual areas of the occipital lobe and the precuneus, regions associated with the processing of visual stimuli and attention for two different packages. On the individual level, a significant activity change was found within the regions of reward processing. Specifically, when people looked at unattractive packages, the researchers found an increased activity in areas of the frontal lobe and insula cortex, regions often associated with processing aversive stimuli such as unfair offers or disgusting pictures. With these results, they explained why attractive packages gain more attention at the retail, and this, in turn, positively influences turnovers of fast-moving consumer goods [263].

A study of Reimann et al. [264] found that aesthetic packages significantly increase the reaction time of consumers in choice responses: among a set of offered products, people chose the ones with well-known brands in standardized packages, despite higher prices. This choice resulted in an increased activation in the nucleus accumbens and the ventromedial prefrontal cortex, according to functional magnetic resonance imaging (fMRI). Such results suggest that reward value plays an important role in aesthetic product experiences. A study of Baldo et al. [244] found that brain scan predicts consumer behaviour much better than questionnaires. Specifically, their study showed that self-report-based methods cannot accurately foretell success, while using brain data, the prediction accuracy reached 80%. They also compared how these two different methods might influence the company gross profit. Simulation based on sales data showed that self-report-based prediction would lead to a 12.1 percent profit growth, while brain scan-based prediction would increase profit by 36.4 percent. Brain data analysis (using the preference index by Davidson [188]) demonstrates that the brain produces significant emotional responses within one second after a product picture (shoe in such case) is presented on the screen. Thus, this innovative neuroscientific approach greatly improves brand image and brings considerable value for organizations, shareholders, and consumers.

A study of Modica and colleagues [260] has investigated the cognitive and emotional reactions to the cross-sensory interaction (sight and touch) with products belonging to different categories. They have found that people have a higher tendency of cerebral approach in response to comfort food during the visual exploration and the visual and tactile exploration phases and towards foreign food products in comparison with local food products. For this latter interaction, also a higher mental effort index (measured as the increment of the theta band in the frontal lobe) has been found.

### 7.4. Service

Services are, by definition, intangible and produced and consumed simultaneously. Also, for services, neuromarketing tools can be used to help researchers evaluate both pre- and postpurchase. With regard to service products, it is possible to affirm that they exhibit no immediate rewards (e.g., home protection systems, insurance policies, and preventative medicine), do not generate much emotional involvement, and, therefore, may receive relatively low processing priority, unless emotional rewards can be invoked. Using fMRI, it was highlighted that when customers think they are being treated unfairly, a small area called the anterior insula becomes active. It means that transactions between a service provider and a service customer are presumed to be based on trust. When trust is high, a hormone called oxytocin fills different areas of the brain. As a result, service marketers could theoretically experiment with different levels of trust to see which one generates satisfying levels of oxytocin given by service production parameters. It would also allow the service marketers to determine how quickly these levels are internalized, meaning the level of trust might need to be increased in order to maintain that sense of pleasure. This information would allow the service product marketer to determine which critical incidents are most damaging, so he/she could plan more efficient and targeted recovery effort for service failure and thus reduce customer loss [265]. Neuromarketing can be used to help service researchers in developing more effective pricing strategies. When customers think they are being treated unfairly, the brain's response is similar to that of smelling a skunk. Such a powerful, negative, and primitive reaction easily overwhelms the deliberation of the more logical prefrontal cortex region. Under these conditions, the perceptions of exchange fairness by a service consumer probably take on even a larger role than first imagined. If unfairness is perceived, it is very difficult to reestablish the relationship as the brain has neural wiring from its early formative period that protects it from known dangers—just as it continues to repeat “*safe*” behaviours. It would be possible to set up experiments depicting various acts of service delivery “*unfairness*.” Neural scanning of the anterior insula would detect and measure the degree of activity generated by various depictions. This information would allow the service product marketer to determine which critical incidents of this type are most damaging, plan more efficient and targeted recovery effort for service failure, and thus reduce customer loss [266].

### 7.5. Pricing

Pricing is one of the key components overseen by companies in positioning their products and represents a fundamental variable affecting the organization's business result and benefits [267–270]. Some scholars have explored the impacts of pricing on shoppers' purchase decision-making and product choices [271]. Despite the amount of academic knowledge available, companies appear to use little of it when setting prices, leading to suboptimal situations for both consumers and companies. Understanding the psychology and neuroscience process of pricing evaluation may be of crucial importance if companies want to get optimal decisions, and recent behavioural research, for instance, has explored mistakes made by consumers when they process prices ending in 0.99 rather than those represented by a whole number: this entails that individuals pay less attention to the last numbers of a sequence [272]. Other research has started to examine the social role of pricing and how individual differences can influence the perception of prices by consumers [273].

A markable phenomenon often observed in price policy is the following: a similar price can be perceived by the shopper in two different ways, depending on diverse product categories: It could be perceived higher when customers consider buying a product at that price as a loss [219]. Instead, high prices can be perceived as an indicator for high quality and can enhance the product value and the probability that customers buy the goods [270, 274]. This is particularly true when customers have uncertainty about buying a product because they are not familiar with it yet. However, asking consumers about pricing issues can sometimes be ineffective. For instance, consumers are often not able to recall prices [275, 276], and it is very difficult for them to specify abstract economic concepts like the “*willingness to pay*” or experienced utility. In addition, they might respond strategically when asked about constructs like price fairness. Knutson et al. [68] examined the neural correlates of the negative price effect. Using an fMRI scanner, subjects in the first task saw a product and then saw the same product with its corresponding price information. In the end, they had to decide whether to buy the product or not. The results resembled those of studies examining the neural correlates of anticipation and the receipt of gains [277, 278] and losses [179]. Hence, the activation of the nucleus accumbens (activation through the anticipation of gains) correlates with product preferences, the activation of the insula (activation through the anticipation of losses) with high prices, and the activation of the medial prefrontal cortex (activation through the processing of gains and losses) with reduced prices. This result supports the speculation that activity changes in the insula might reflect the perception of a loss and, thus, the neural representation of a negative price effect [219]. This information can be important, for example, in identifying the price limits. Another important issue in price policy is the opportunities to customize the pricing for a specific customer segment. For this, it is necessary to know how people calculate their individual “*willingness to pay*” (WTP)—the maximum price that a buyer is willing to pay for a specific object [279]. As mentioned above, the determination of this abstract concept is very difficult with the current research methods. An fMRI study conducted by Plassmann et al. [48] observed that hungry subjects, while placing bids for the right to eat different foods in a Becker–DeGroot–Marschak auction [280], offered a specific amount of money that suited the subject's personal WTP. The results suggested that the activity in the MOFC and in the dorsolateral PFC encodes subjects' WTP for the items supporting the hypothesis that the medial orbitofrontal cortex encodes the value of goals in decision-making.

Another relevant matter is the correlation between price and satisfaction. In 2008, a study performed by Plassmann and colleagues [47] demonstrated that the MOFC activity is influenced not only by sensory components but also by cognitive stimuli (e.g., the price of an item). In their fMRI study, they asked participants to drink three different Cabernet Sauvignon wines. The subjects were told that they would be sampling five different wines, identified by their retail price—$5, $10, $35, $45, and $90, respectively. The study was manipulated, unknown to the participants, and the $5 and $45 bottles of wine and $10 and $90 ones were identical. When people tasted the same wine twice in raw (unconsciously), the MOFC area was increased when they thought to enjoy the more expensive vintage. Similarly, the effect of price on MOFC activity was higher for the cheaper wine over the more expensive wine. Hence, this suggests that the effect of a price increment on MOFC activity might be relatively great at low prices compared with high prices. In contrast, no price effect on primary taste areas was reported because the cognitive processes that encode both the flavour expectancies and the sensory properties of the wine are integrated in the MOFC. Additionally, the flavour expectancies determined by the price change do not influence the sensory representation. The conclusion of Plassmann's work was that the MOFC, an area that modulates the hedonic experience of flavour and taste, is itself modulated by intrinsic qualities of a consumed item and by extrinsic factors, such as expectations and price.

In 2013, Kai Müller, who was working in Germany, in the Neuromarketing Labs, performed the “*Starbucks study*,” one of the most mediatized studies on pricing. Subjects' brain waves were recorded via EEG and indicated when the price was right. Kai Müller developed a way to measure brain waves and hit upon feel-good prices. He stated overtly that “*classic market research doesn't properly work*,” adding that people usually involved in market researches cannot always be trusted to honestly state how much they would be willing to pay for something, while it is harder to fool such neurophysiological techniques. There is a region in our brains that monitors proportionality. When proportions are radically off, for example, when a cup of coffee costs 10¢ or $100, this region sends an alarm. In Müller's Starbucks study, an undisclosed number of subjects were shown several images of the same cup of coffee, each paired with a different price tag. Judging by neuroimages, he concluded that Germans would happily pay 33% more than the current price for a small cup of Starbucks coffee. If he is right, Starbucks would be missing out on a whole slice of profit. In a follow-up experiment, Müller tried to understand how much people are willing to pay for a coffee. Specifically, Müller and his staff, at the Munich University of Applied Sciences, installed a caffeine vending machine that dispensed coffee for 70¢, cappuccinos for 80¢, and then left students to pick an appropriate price for macchiato. After several weeks, the macchiato price levelled off at 95¢. When Müller performed his neuropricing lab experiment, he found out that subjects' brain waves also indicated 95¢ as the ideal price for the vending machine's macchiato, thus exactly matching people behaviour. These experiments appear very interesting because the methods look affordable and not too much difficult to be applied, being about an EEG tool.

### 7.6. Brand

**Figure 6:**
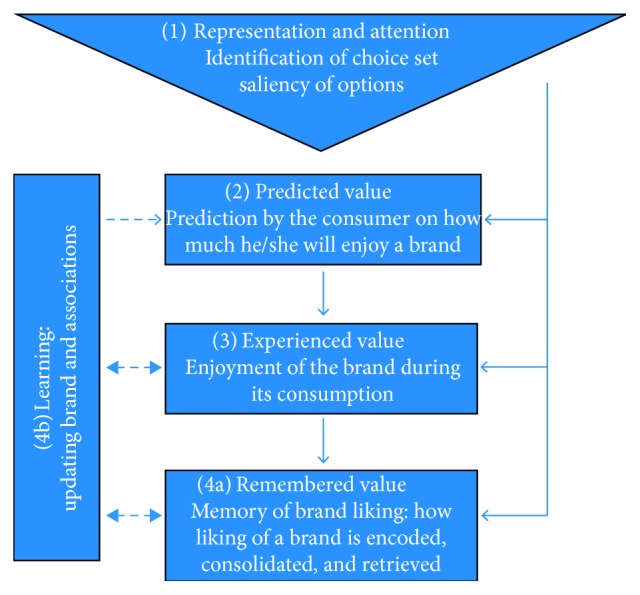
Value signals important for brand decisions [62].

A brand refers to the identity of a company. It represents the products or services a company offers, highlights their quality, and can help create a follower base for the latter. To succeed, a brand must be recognizable, steady, and specific and must fit or support the company's products and services. The application of neuroscience to the consumer psychology of brands has gained popularity over the past ten years within the academic and the business world. Plassmann et al. [47] reviewed the previous neuroscience work relevant to the understanding of underlying brand decision processes. They structured the review using a simple framework for consumer decision-making (Figure 6) based on previous works in consumer psychology [281–283].

Moreover, as shown by several recent reports during the past few decades, the search for unconscious processes and implicit measures of branding is an active field of inquiry in consumer psychology [284–299].

One central topic of brand research is whether the consumer's decisions are influenced by brand information. Deppe et al. [300] addressed this question in a study designed to determine which neural processes are involved in the brain during the processing of brand information [219]. In their fMRI study, participants were asked to make fictitious buying decisions between two similar products that were differentiated only by brand information. In one part of the study, subjects had to choose between the brand with the greatest market share—which had been declared as the target (T) brand in the preliminary phase—and diverse (D) brands (TD decisions). In the second part of the study, they had to decide between two diverse brands (DD decisions). The data analysis showed a significant difference in brain activity between the TD and DD decisions, if the subjects had declared the target brand as their preferred brand (first choice brand (FCB) group) in the pretest phase. A closer look into the brain activities of the FCB group showed a reduced activity in the DLPFC, left premotor area, posterior parietal cortex, and occipital cortex—areas that are generally associated with working memory, planning, and logic decisions. Deppe et al. [300] assumed that, for decisions comprising the favourite brand of the consumer, strategic processes are no longer relevant. The responsible brain region is deactivated, and a “*cortical release*” occurs [301]. In contrast, an increased activity was measured in the VMPFC, the inferior precuneus, and the posterior cingulate cortex. These areas operate as association cortices and have important functions in combining incoming information with background knowledge, as well as the recall of episodic memories, and self-reflection. The increased activation in the ventromedial prefrontal cortex during decisions in the FCB group could be interpreted as the integration of emotions into the decision-making process [81, 219].

The results, therefore, revealed a so-called “*winner take all*” effect: only the subject's favourite brand can emotionally move the decision-making process—the finding is crucial for marketing research because it runs against the well-established concept of the consideration set.

While the consideration-set theory assumes that there is a set of goal-satisfying alternatives [302], the results of Deppe et al. provide evidence that only the favourite brand can trigger significant cortical activation patterns. Intriguingly, a lesion study conducted by Koenigs and Tranel [303] confirmed the suggestions of Deppe et al. [300]. People with damage within the ventromedial prefrontal cortex that exhibits irregularities in emotional processing did not show the normal preference biases when exposed to brand information. Plassmann et al. [304] provided additional support for the investigated “*first choice brand effect*.” Their study aimed to explain the influence of brand information within uncertain situations, by investigating the role of the prefrontal cortex during decision-making under risk. The subjects participated in a brand choice task where they had to choose between sixteen travel brands, for travel to a risky and a less risky destination. In addition to the “*first choice brand effect*,” the data analysis exhibited a more prominent activation of the medial prefrontal cortex when the subject faced risky decisions. Plassmann et al. reasoned that the integration of emotions in the decision-making process, as opposed to analytical decision strategies, is of particular importance in risky decision-making. One potential reason for this might be that emotions could provide additional conscious or unconscious information.

Finally, neuroscientific tools can be used to evaluate the rebranding process. Rebranding is costly and time-consuming, and as the number of corporate rebranding practices increases, the failure rate is high compared to the successes [305, 306].

A rebranding exercise for a new logo is a key task for a big company. Kapferer [307] also agreed that brand transfers would pose risks such as loss of choice, loyal customers, and market share. So, it is very important to do several studies to design a new logo. In 2016, Telecom Italia (the main and historical telecommunication company in Italy) launched an initiative after three years of strategic study which focused on transforming its brand into a combined service brand (that was previously separately positioned as Telecom Italia and TIM).

The company chose to maintain one of the two previous brand names, TIM, but with a new graphic icon refined through traditional marketing research methods combined with a neuromarketing test. The company performed a long process of selection among large numbers of different logo ideas, over more than two years. Ultimately, two alternative symbols made the short list. A final-stage research study with two different methods was planned: quantitative surveys with a predefined questionnaire (web-based interview) and a neuromarketing test (ET, GSR, HR, and EEG). Combining the results of quantitative surveys with a neuromarketing test, the latter provided the insight to aesthetically refine and strengthen the impact of the final version which in the final decision-making process has been firmly endorsed by top management and become the logo selected for the renewed TIM brand [308].

### 7.7. Web Usability and Apps

During the last twenty years, the Internet has grown quickly in usage and penetration: this has led to changes in the behaviour of people, business, and organizations. Nowadays, it is almost ordinary to use the web daily, to look for any sorts of information, to get products and services, and to socially interact through different platforms and websites. Hence, companies and organizations make their best to obtain a position within this network in order to attract and retain users and customers. To achieve this objective, it is necessary to have an interesting and effective presence online, by having more engaging websites than the competitor ones. To do this, it is very important to have specific knowledge about the needs of potential users and customers, alongside with the ability to establish personalized services that satisfy these needs [309]. This is directly related to how people interact with the websites, how they behave in browsing the Internet, what their preferences are, and what areas drive their attention: these concepts are encompassed by the notion of web usage mining [310, 311].

Researchers and website developers, since the birth of the web, have kept always in mind a focal question: What are the optimum structure and content of a website for attracting the web users' interest and preferences? [312]. The answer is not easy, and many efforts have been developed over the years. Traditionally, web user behaviour on the web has been studied by using web usage mining techniques [313, 314], where the web log files, which contain records of web user activities, are processed to extract information and knowledge about their navigation and content preferences. This information is used for improving the site structure and content with the aim to provide online navigation recommendations through an automatic recommendation system [315].

The adoption of new technologies, like ET, EEG, HR, and GSR, also in the web area, is aimed at better understanding what kind of people look at and pay attention to the website and the characteristics of the user experience.

There is a large set of studies aimed to link a web user choice with different variables or behaviours. For example, in 2006, Chandon et al. [316] (for more details, read a review of eye-tracking research in marketing [317]), performed an eye-tracking experiment that analysed object choice situations associated with brands. They concluded that visual attention is relevant in a user's choice process, suggesting that those objects with low choice probability could be enhanced if they were placed next to the objects with high choice probability. Another study was performed by Krajbich et al. [318] with the objective of relating choice process with gaze position.

To study the effect of faces for the visual appeal, efficiency, and trustworthiness on the website, Djamasbi et al. [319] conducted an ET study, and they discovered that users believe that pages containing images of people's faces are more appealing and that it is easier to perform tasks in them, as opposed to those that do not contain them. Furthermore, the analysis revealed a strong positive correlation between trusting the informational content of a page and its visual appeal. In 2010, Lee and Seo performed a usability study in which typical techniques were mixed with biosignal analysis [320] finding that using new technologies (EEG and ECG), it is possible to obtain a reasonable and valuable method for web evaluation, since they obtained 70% precision with respect to traditional methods (user performance measurements, keystroke analysis, satisfaction questionnaires, and interviews). In 2011, Reutskaja et al. [321] using the ET tool studied the user's behaviour when choosing between objects under conditions of time pressure and overload. The results highlighted that objects placed in the centre of the screen have a higher probability of being chosen than objects with similar characteristics placed in other screen zones. Khushaba et al. [322, 323], using EEG and ET, studied the user's preferences to find interdependencies among the EEG signals from cortical regions in a decision-making environment and also a way to quantify the importance of different product features such as shape, colour, or texture in these decisions. The results have shown there is a clear and significant change in the EEG power spectral activities that take place mainly in the frontal, temporal, and occipital regions, occurring when participants indicate their preferences.

Using pupil dilation and EEG, Slanzi et al. [311] explored the user's behaviour from a physiological perspective, to assess the choice represented as a click intention. They found the when people choose and click, they have greater pupil size. These results show that it is possible to create a classifier for web user click-intention behaviour based on merging features extracted from pupil dilation and EEG responses.

Moreover, in recent years, smartphone APP design gets more attention from both industrial companies and academic researchers, which brings us not only great surprise but also abundant convenience to our daily life [324, 325]. An increasing amount of research has been conducted to determine what business opportunities a mobile presents as an added channel for driving revenues by e-commerce. The advantages and disadvantages of APP design affect the user experience actually directly, and smartphone apps' large commercial interests encourage firms to continually improve the performance of apps [326] with regard to benefits and obstacles to their user experience and their engagement. Recently, the evaluation of the user experience of smartphone apps was mainly based on the perspective of a relatively simple, objective evidence-less evidence-based questionnaire [327–329]. Quing-Xing Qu et al. [330] in their study proposed that eye-tracking data could be used as objective criteria to evaluate user experience for smartphone apps. A correlation model between smartphone APP design variables and user experience was built based on Quantification Theory I [330, 331]. The app's performance, in terms of emotional engagement and attentional activation, drew attention as early as 2013, when in a study Adhami [332] used neuromarketing technology to evaluate three different apps, for determining what drives users when browsing, selecting, and purchasing items. He discovered the following: (1) apps have a significant impact on overall brand perception, (2) user experience impact on whether or not the user makes a mobile transaction, and (3) there are some elements that make a difference to the user in a mobile transaction.

Finally, the use of web advertising to promote brands, products, and services is another important aspect. Often, an enormous problem with online advertisements is that Internet users tend to avoid them, leading to ineffective branding campaigns and a significant waste of money for advertisers. In response, more advertisers use technology to measure the visibility of advertising campaigns on the website of publishers [333]. Programmatic advertising and the ability to collect data on consumer and ad impressions allow advertisers to automate the buying and selling of ads and to achieve an effective personalized targeting of audiences. In such a case, online advertisements are considered in a better position with respect to the TV and print media to estimate how successful a particular ad is in driving a purchase decision or in raising brand awareness over time. However, the promises rest on the assumption that the served ad impressions are viewable by Internet users; that is, “*contained in the viewable space of the browser window, on an in-focus browser tab, based on pre-established criteria such as the percent of ad pixels within viewable space and the length of time the ad is in the viewable space of the browser*” [334]. Viewable in this context refers to the user's opportunity to see an advertisement, regardless of whether they have seen it. This simple assumption is however challenged by companies such as Google, Comscore, and Nielsen that daily analyse billions of impressions from campaigns over thousands of publishers and observe that most of the served impressions are actually never seen by Internet users. A well-known commented statistic released by Comscore in 2013 indicates, for instance, that half of the publishers' list is not seen by users. In 2016, the percentage of ads being seen by people in most of the countries around the world is still relatively low, between 40% and 50%. Also, Facebook, the most popular social network, that attracts a large part of ad investments is also criticized: according to the Business Insider article, “*Facebook ads are far less viewable than advertisers were expecting*” [333].

For these reasons, in the last years, a new way to make advertising has gained attention: the *native advertising or sponsored content*, which is considered a means for advertisers to cut through the clutter and for online publishers to boost their diminishing ad revenues [335]. It refers to any paid advertising that takes the specific form and appearance of editorial content from the publisher itself, and compared to display ads, which users avoid or ignore, it has more opportunity to be noticed [336–338].

Native advertisements offer a higher level of reader attention to marketers. The visual focus is more on banners than on native advertisements, especially on the text. This direct focus on the advertisement can help the reader form associative networks of words and brand assets that influence and strengthen brand perceptions subconsciously. The main problem is that there is an almost endless amount of content, but only a limited amount of time to consume everything. A marketing research conducted by Nielsen in 2014 revealed that mobile users are less open to ads. Only 13% of Americans using mobile devices say they are willing to receive ads on their phones in exchange for services. And yet, mobile advertising can often be a great advantage for advertisers. Studies have shown that exposure to mobile ads can produce a 45% lift in intent [339].

The most recent research by Neurons Inc. has revealed that mobile advertisements trigger customer reactions in less than a second (i.e., 400 milliseconds) [340, 341]. Thus, data suggest that desktop advertisements take 2-3 seconds in comparison with 400 milliseconds for mobile advertisements. Therefore, these findings reveal that companies should rearrange their strategies in order to gauge customer attention. Indeed, winning customer and mobile users attention can be valuable prospects.

But how does an advertiser get his audience to pay attention to their advertisements on mobiles? And how can that attention be measured? The click-through rate (CTR, or the ratio of clicks to impressions) only tells a fraction of the story. Even the highest-performing mobile advertisements—those with click-through rates above 1%—still fail to convert the other 99%, which could amount to millions of unconverted impressions over the course of a campaign. Very little is being done to understand the value of these impressions because there are very few options to measure it.

Therefore, the modern mobile marketers face a dilemma: How can they ensure that their advertising receives customer attention it deserves and maximizes the value of each impression?

In 2015, Nielsen conducted a study for Sharethrough, a software company that enables leading websites and apps to manage their in-feed, native ads. To understand the effectiveness of mobile advertising, the study compared native advertising and banners, both placed in feed. Using a combination of EEG and ET, Nielsen quantified where and how participants' focus was being directed. The main findings were that (1) native advertisements appear to receive two times more visual attention than banners, (2) banners are processed peripherally, (3) native advertisements are being read, (4) headlines of native advertisements can be optimized to trigger associations, and (5) brand assets impact brand resonance lift.

In conclusion, as mobile adoption and usage grow, consumers' attention will become increasingly elusive. Native advertisements command focus and attention. They can be an effective method for marketers to share their brand's stories and narrative to the highly distracted mobile consumer [339], and the use of the new technologies could be considered very useful to measure the impact of these advertisements.

### 7.8. In-Store Retail

The consumer perceives the store environment by all five sensory organs. Solomon and colleagues [342] describe this perception as the process in which people collect, organize, and interpret information from the outside world. In a store, several elements such as light, sound, smell, and merchandising influenced the consumer behaviour during shopping. Specifically, these elements impact on the retail atmosphere and have an influence on consumers' emotional involvement. They are considered important marketing tools due to the fact that they increase retail space and enable easier orientation for the customers, making them feel comfortable in the store. Otherwise, it is possible that the customers would be discouraged from buying goods that they would be interested in a different situation [343]. Many facets are overlaid in the overall creation of the atmosphere, which affects both subconsciousness through senses and a specific state of mind for customers [344]. The moment when customers make their decisions is significantly affected by what they see, hear, smell, and touch in their surroundings because these are immediate signals for the creation of emotions [345]. For the consumer decision system, emotions and feelings are the primary medium [346].

Importantly, retail market is changing at an incredible speed, and therefore, every retailer is trying to adopt innovative ideas that can help differentiate them from competitors [347].

The most critical requirement in modern retail stores is a high quality of lighting, which increases the image of these stores, attracts the potential customers, points their attention to products being offered, and finally raises the sales [348]. By using correct lighting (intensity, colour temperature, and illumination angle), it is possible to attract customers' attention, create a unique in-store environment, and encourage customers to stay longer and come back to these stores. Moreover, light is a tool that can be precisely controlled and measured. The consumer's interest for visual stimuli is due to the fact the visual sense is the most developed and predominant in the human brain. Almost a quarter of the human brain is involved in visual processing, much more than in case of any other sense. Indeed, the human eyes contain approximately 70% of body sensory receptors. The simplest and the most successful way to reach customer attention in the food selection process is through visual (products) illuminated in an eye-catching way. In the case where visual and musical stimuli are presented simultaneously, the brain puts more credibility and impact on the visual part.

In 1994, Areni and Kim [349] conducted a study in which they found out that brighter indoor lighting of the store makes a more positive impression on consumer perception reflected in time spent looking at the goods. Total preferences of lighting and colour temperature (chromaticity temperature) which is produced can be changed depending on weather and moods of customers [350].

A significant attribute of marketing tools in neuroscience research and visual merchandising is that 50–80 percent of unplanned purchases are influenced by initial (positive) neural excitement during shopping in-store units. Thanks to the innovative interdisciplinary approach with the use of neuromarketing, efficient marketing strategies can be developed and human emotions can be stimulated by managing a higher number of closed contracts, increasing incomes and improving the ability to buy [351]. Understanding the needs, purchasing behaviour, and changing lifestyle of today's shopper is critical in being able to deliver on their immediate and future needs. While it is true that shoppers' decisions are no longer limited to the in-store ones, POPAI's 2012 Shopper Engagement Study found that nowadays more than ever, shoppers are making an overwhelming number of their purchasing decisions in stores. In fact, the in-store decision rate has climbed from 70% in 1995 to 76% in 2012. With the advent of smartphones, shopping apps, mobile coupons, and other innovations, the shoppers' path to purchase is considerably different today in comparison with the past. Usually, the decision-making process of shoppers does not occur until they see a product in the store. Therefore, how a product is displayed in a store and is supported by in-store marketing materials can often be instrumental in leveraging sales. In the study conducted by POPAI in collaboration with Sands Research, the EEG brain signals and ET fixations have been recorded, during the shopping experience in a store. The main findings were the following ones:

1. The items placed in the cart by a shopper produce a positive emotional brain response, thus demonstrating that there is a brain response capable of predicting a purchase.
2. Subsequent eye fixations to the to-be-purchased item show the purchase intent effect, but it is diminished. This means that the biggest reward comes earlier in the discovery process [352].

Cherubino and colleagues in 2017 [77] investigated the brain activity (through the EEG) and the eye gaze (through the ET) of some individuals who were experimenting a visit to specific areas of a supermarket; they were focused particularly on the purchase of some products in the fruit and vegetables department. The results show how neurophysiological tools could be used to get kinds of information that would not be obtainable otherwise with the verbal interviews. The main findings demonstrated that the elements that made the individuals' shopping experience more enjoyable are the following:

1. An innovative packaging and countless graphic customizations
2. A better organization of the shelf (allowing an easier customer experience with a low value of the mental effort index)
3. The presence of the farmer outside the store

These elements returned higher neurometric values of pleasantness in the experimental participants.

### 7.9. Neuropolitics

Since the last decades, the emergence of political neuroscience has meant “*before*” and “*after*” in the way of developing and analysing the approach between neuroscience and politics, in the realization of new research studies which allow to understand the impact of the electoral messages of each of the parties and candidates in the population, in order to predict the outcomes of the electoral process and success. Neuropolitics is defined by Antoni Gutiérrez-Rubí, a political consultant, as a “*new discipline capable of understanding the brain of people in their capacity as citizens, voters or activists that allows knowing and understanding how it works, how it articulates images, values, feelings and channels its decisions*” [353]. That is, neuropolitics includes a set of techniques aimed at knowing the electorate so as to predict its behaviour, as well as the design and elaboration of communication campaigns meant to seduce it [354]. With regard to the “*political brain*,” a series of studies highlighted that political information is recorded at different brain levels.

Kanai [355] conducted a study in which it was discovered that, at a physical level, there are differences in the brain of conservatives and liberals and, therefore, a difference between cognitive systems. Using fMRI and according to different tests, the researcher found that the more liberal people had more volume of grey mass in the cortex of the anterior cingulate, while the more conservative people had more volume of grey mass in the right amygdala of the brain [355]. Therefore, the liberals presented more brain activity in the region than conflicting process information, and 10% of the participants with this orientation were more predisposed to rectify a response incorrect than the conservatives [356] (Braidot stated “*What happens in the brain of the electorate, what happens in the brain of the candidates?*” (p. 7 in [357]). According to the theory of affective intelligence, the most important emotions for political behaviour are enthusiasm (the opposite of depression) and fear (the opposite of calm). Thanks to different tools of neuromarketing, it is possible to analyse how effective a political campaign is and how it can influence votes for candidates from an unconscious level. Neuroscientists are familiar with the established research evidence of more than 50 years that personality variables accompany differences in political opinion [358].

A brain imaging study of swing voters, in the summer of 2007, was analysed by a group of researchers who published it in The New York Times on November 11, 2007 [359].

Politicians use many different techniques to hold and extend their appeal to their traditional voters. It is not surprising that political campaigners also try to benefit from this knowledge as we learn more about how the human brain works.

Westen [360] in a study based on brain scanning found that “*the political brain is an emotional brain. It is not a dispassionate calculating machine, objectively searching for the right facts, figures and policies to make a reasoned decision*.” He built this formulation by analysing political TV advertisements that, whilst banned in the UK, were widely used in the US. Indeed, these advertisements represent the items that received most of the campaign budget of the candidates (about some millions of dollars). Westen concluded that “*Republicans understand what the philosopher, David Hume, recognized three centuries ago: that reason is a slave to emotion, not the other way around. Except for the Clinton era, Democratic strategists for the last three decades have instead clung tenaciously to the dispassionate view of the mind and to the campaign strategy that logically follows from it, namely one that focuses on facts, figures, policy statements, cost and benefits, and appeals to intellect and expertise*” [361].

Vecchiato et al. [362] used EEG technology to assess the Italian Prime Minister's TV speech in 2009. The brain activity was observed in two groups of people divided into swing voters and Italian Prime Minister's “*supporters*.” The results showed a different brain activity between two groups: for the supporters, a greater power spectral activity was observed throughout the speech than the swing voters, who were less attracted by the speech.

### 7.10. Neurotaste

Neuromarketing has been widely used in the food and beverage sector. In recent years, researchers have focused their attention on applying the neuroscientific methods not only to the extrinsic features of the food and beverage sector products (i.e., packaging, price, shape, colour, and texture) but also to their intrinsic values: flavour/taste and scent/aroma. This kind of study named “*neurotasting*” includes the concepts such as “*neurogastronomy*” [363] and “*neuroenology*” [364]. The taste is a vital sense in humans because of its active role in regulating nutrition or avoiding harmful substances [90]. Information conveyed via the gustatory system aids in identifying edible and nutritious foods, makes the humans able to avoid toxic substances, and drives the hedonic evaluation of nutrition, which can take place before, during, or after ingestion. For such a reason, the interest in understanding the cognitive processing related to the human sense of taste grew up during the last decades, not only for basic research on food and nutrition but also for the clinical applications and consumer industries. Perception of the basic tastes of sweet, salty, umami, sour, and bitter as well as the oral sensation of fat plays a vital role in determining food acceptance, preference, and choice. Our subconscious state associates certain foods with pleasure and happiness, and certain others with fear [365]. The pleasure we derive from eating, termed “hedonics,” provides us with the drive to consume a food. Taste is not simply defined by our genetics but can be modulated by a variety of biological and environmental factors, including body mass index and the consumption of certain foods [366, 367], smoking and alcohol consumption [368–370], aging [371, 372], gender [372], and exposure to pathogens [372].

There are numerous neuroscientific studies investigating the relationships among communication, perception, and satisfaction experienced by consumers [373].

Using fMRI and MEG, it has been possible to determine the dynamics of the human brain processing of information coming out from the gustatory system. In particular, the human insula has been associated with the initial sensory processing of taste [374]. It is hence commonly considered the primary taste area. The orbitofrontal cortex (OFC) and prefrontal cortex (PFC) have been linked to the processing of hedonic aspects of taste [375] and are often regarded as the secondary taste area. Several studies based on less invasive technologies, in particular fNIRS and EEG, confirmed the theory about the important role of PFC in decoding information related to the taste [376, 377]. These findings couple with the widely accepted theory about the relationship between the human PFC activity and the motivational processes towards sensorial stimuli based on which an increasing left hemisphere activity is associated with approach attitude, while an increasing right hemisphere activity is associated with withdrawal attitude [188]. It is important to remember that the first neuromarketing study conducted by Read Montague (and described in the previous part of this paper) was based on the taste perception, which is important in order to understand consumers' preferences about common beverage products such as Coca-Cola and Pepsi [99]. In Plassmann and colleagues' study [47], participants have been scanned with an fMRI while they were performing a wine tasting and preference rating task. Various food products and beverages, such as chocolates, wine, and cola, have been administered in the fMRI scanner. These products are particularly easy to administer through a computer-controlled pump attached to a tube that delivers controlled amounts of fluid into the participant's mouth.

An important application of the neuroscientific methods could be to better understand how the smells influence the consumers in the food choice [90]. In fact, odours and tastes can lead to specific memories: some people may have particular odours associated with friends, family members, or other loved ones, and these memories can be automatically triggered by a brief exposure to the same odour [340]. The importance of the variable “*olfaction*” during the tasting experience had already been naturally demonstrated when people are recommended not to drink wine when they have cold (the closed nose decreases the pleasure of drinking). This has also been empirically demonstrated in a couple of studies [197, 198] with an experimental protocol taking into account an EEG index, assumed as an indicator of approach or withdrawal (AW) motivation [188], and an autonomic index (emotional index (EI)), deriving from the matching of the heart rate and galvanic skin response activity [61]. The experiments have provided the degustation of two types of Italian wines, and the process has been divided into two phases as well: smell and tasting. For the tasting phase, two different conditions were considered: “*with open nose*” and “*with closed nose*.” The results of both research studies showed an impact of the smelling phase on the emotional index in comparison with the other two phases of tasting (with and without olfactory component) and a trend of major approach attitude in correspondence of wine tasting with the olfactory component (in comparison with the other two conditions).

To conclude, these techniques could be applied not to encourage junk-food addiction but to make healthy food more attractive, appealing, and thus more consumed.

### 7.11. Neuroaesthetics

As Santayana [378] observed, “*Humans are drawn to the aesthetic features of objects and the environment around them*.” Such features are not mere inconsequential adornments; they influence people's affective responses, decisions, and behaviour. In fact, aesthetics plays a central role in consumers' choice of products [264, 379], in judgments of artificial [380, 381] and natural environments [382, 383], and in attitudes, judgments, and behaviour toward other people [384–386]. Some of the questions that neuroaesthetics aims to answer are related to the understanding of which neural processes of aesthetic features influence people's attitudes, decisions, and behaviour and, in general, what are the neural underpinnings of aesthetic appreciation [387].

Neuroaesthetics is an emerging discipline, within cognitive neuroscience, that investigates the biological foundations of the aesthetic experiences [388]. The discipline merges empirical aesthetics with cognitive and affective neuroscience [389]. “Neuroaesthetics” is a term coined by Zeki [390] and refers to the study of the neural bases of beauty perception in art [391].

At its core, neuroaesthetics is a certain way of doing aesthetics, using neuroscience as a method of investigation where other aesthetic approaches have used philosophical analysis or psychological models. Neuroaesthetics, therefore, studies how the brain processes support the aesthetic behaviour. Although the real experimental work on this issue has only begun in the last 20 years or so, the conceptual inclination to investigate the neural mechanisms underlying the aesthetic behaviour can be traced back to the eighteenth and nineteenth centuries [392].

Neuroaesthetic studies are usually performed recording the cerebral hemodynamic responses, with fMRI for the observation of computer screen reproductions of paintings or sculptures (reviewed in [391]). Neuroelectrical and neuromagnetic correlates of such brain activity were also addressed by few authors by using MEG [393] and EEG [394, 395] brain imaging modalities. However, in all the published scientific reports related to the study of brain activity with fMRI, MEG, or EEG modalities, the fruition of the paintings or the sculptures was made possible to the subjects through a presentation of a series of images of such fine art works on a screen. This was due to the fact that both fMRI and MEG are not portable. On the contrary, modern EEG technologies allow to record the brain activity in different environment and mobile conditions, for example, during the fruition of real masterpieces in a fine art gallery environment, where they are usually observed by visitors [193].

The study of neuroaesthetics has mostly dealt with aesthetic appraisal: in this context, participants are usually asked to explicitly judge a visual stimulus as either beautiful or ugly. Kawabata and Zeki [396] used fMRI to investigate the neural correlates of beauty perception during the observation of different categories of paintings (landscapes, portraits, etc.) that were judged by participants as beautiful, neutral, or ugly. The core imaging results revealed different brain activations for judged-beautiful stimuli versus both judged-neutral and judged-ugly images in the medial orbitofrontal cortex (OFC). The differential activation observed in the OFC consisted of decreased activity with respect to baseline, with judged-ugly stimuli evoking the lowest level of activation. Using a similar methodological approach, Vartanian and Goel [397] carried out an event-related fMRI study, in which explicit aesthetic preference for representational versus abstract paintings was investigated in three stimulus versions: original, altered, and filtered. Participants indicated their preference with a button press at each stimulus presentation. Representational paintings evoked higher preference than abstract paintings. In both categories, original paintings elicited the highest preference. In 2009, Cela-Conde et al. [393] investigated gender-related similarities and differences in the neural correlates of beauty by using a set of images of either artistic paintings or natural objects, divided into five groups. Through MEG, it was possible to detect an enhanced activation for “*judged-beautiful versus judged-ugly*” stimuli in several parietal foci (bilaterally for women and mainly in the right hemisphere for men) with a latency of 300 ms after stimulus offset. The activation of parietal areas during aesthetic experience was also shown in an fMRI study by Cupchik et al. [398], in which participants viewed various categories of representational paintings that were classified as the “*hard edge*” (containing well-defined forms) and as the “*soft edge*” (containing ill-defined forms). Enhanced activation of the left superior parietal lobe was observed for the “*soft-edge*” paintings, particularly during the “*aesthetic*” condition. Involvement of parietal and premotor areas in aesthetic experience was observed in the fMRI study of Jacobsen et al. [399]. Participants had to make an aesthetic assessment of abstract geometric shapes, the symmetry and complexity of which had been manipulated. Behaviourally, symmetry has been shown to have a strong effect on aesthetic judgment and aesthetic judgment tasks compared to the control condition (observation of the arrow), and activation in areas that serve visuomotor processes, including the intraparietal sulcus and ventral premotor cortex, has been enhanced under both conditions. In 2007, Di Dio et al. [400] carried out a study in which two versions of Classical and Renaissance sculptures were presented: original and proportional. The image results showed that some lateral and medial cortical areas (lateral occipital gyrus, precuneus, and prefrontal areas) and, importantly, the right anterior insula were activated by the observation of original sculptures related to the modified ones. Activation of the insula was particularly strong during simple observation condition, in which the brain could be said to respond most spontaneously to the presented images, and support for this finding comes from the study of Cupchik et al. [398] in which the observation of representational paintings under the “*aesthetic*” condition versus baseline condition elicited bilateral activation of the insula. It is interesting to note that, in this study, no explicit behavioural responses were required in the scanner and that implicit “*aesthetic attitude*” was induced in the participants by specific instructions provided prior to the scanning [391].

As previously said, through the portable technologies, it is possible to conduct the research in different real environment and in mobile conditions. Babiloni et al. [193] conducted a study during a visit to a real fine art gallery, in which they examined how motivational factors as indexed by EEG asymmetry over the prefrontal cortex (relative activity of the left and right hemispheres) could be related to the experience of viewing a series of figurative paintings. The results suggested a strict correlation of the estimated EEG asymmetry with the verbal pleasantness scores reported by the subjects and an inverse correlation of the perceived pleasantness with the observed painting's surface dimensions.

In 2014, Babiloni et al. [401] conducted a study in which they collected the neuroelectrical brain activity, heart rate, and galvanic skin response correlated with the observation of the real sculpture of Michelangelo's Moses within a church in Rome. The group observed the Moses sculpture from three different perspectives, each revealing different sculpture details. In addition, the light conditions related to the sculpture's specific observation were explicitly changed at each location. The results showed that the subjects' cerebral activity varied significantly across three different views and did not have a light condition. In addition, the estimated emotional involvement of the entire population has been higher for the point of observation in which the face of Moses was directed to the observers' eyes. Finally, the cerebral appreciation of the investigated group was found to be maximum from a perspective in which all the details of the sculpture could be easily seen.

Babiloni et al. [192, 402] measured the neuroelectrical and eye movement activities in a group of participants during their visit to a fine art gallery where a series of masterpieces of the Italian painter Tiziano Vecellio were shown. A mobile EEG device with an eye tracker was used for this experiment. The results showed that, in the examined group, the approach-withdrawal (AW) index was significantly higher during the observation of portraits rather than during the observation of the religious subjects. Interestingly, the average AW index estimated in the first 20 seconds of the observation of the pictures remained highly correlated with the AW index evaluated for the second part of the data for all the pictures examined. In addition, the number of eye fixations performed by the subjects in the first 5 or 10 seconds of observation of the most appreciated pictures is significantly higher than the number of eye fixations performed on the pictures that subjects did not like. But such a difference vanishes if the entire period of observation of the pictures becomes one minute.

Importantly, Cartocci and colleagues [403] conducted a pilot study using the neurometric index during the listening of selected pieces of Dante's Divina Commedia in 2016. Half of the participants had a literary formation, while the other half of them were attending other kinds of university courses. The findings revealed that the “*humanist*” group reported higher approach-withdrawal and emotional index values when compared to the “*nonhumanist*” group sample.

Finally, in 2017, Maglione et al. [196] estimated the cortical activity correlated with the perception and appreciation of different sets of pictures by using the neuroelectrical brain activity and graph theory methodologies in a group of original pictures of Titian's and a contemporary artist's paintings (Orig dataset) plus two sets of additional pictures. These additional datasets were obtained from previous paintings by removing all but the colours or the shapes employed (Colour and Style datasets, respectively). The results suggest that the verbal appreciation of the Orig dataset when compared to Colour and Style ones was mainly correlated with the neuroelectric indexes estimated during the first 10 s of observation of the pictures.

## 8. Ethical Issues

As we have seen in the previous sections, neuromarketing is a new field in consumer research that is rapidly emerging. For some observers, the mysteries of consumer choice and behaviour in the human brain are finally unlocked by the “*Holy Grail*” of research technologies. For others, the root of all evil will ultimately give marketers and advertisers ultimate control over our minds and wallets. The truth lies somewhere in the middle, as with most exaggerations. Neuromarketing does bring some quite powerful insights and techniques into the consumer research domain. Actually, the neuromarketing research is not a discipline for turning people into “*zombie consumers*,” but it is a marketing or market research activity that uses the methods and techniques of brain science to better understand the consumer behaviour. It is a distinctive approach to market research because it is based on new knowledge from the brain science and it does not represent a tool to identify the consumer's “*buy button*”, as it has been stated in some contexts. In many cases, neuromarketing is not well understood, but it is controversial [404].

For all these reasons, researchers should be aware and careful with ethical aspects when using brain scans and neuroscience advances to understand consumers' decisions. So, the ethics of neuromarketing is also an issue. Of course, the use of scientific technology to promote commercial interest is not inherently problematic. However, the use of technology that tests the inner workings of the human brain, especially beyond what can be disclosed in traditional behavioural tests, raises significant ethical problems. These problems fall into two main categories: (1) protection of different parties that may be harmed or exploited by neuromarketing and (2) consumer autonomy protection [405]. In someone's mind, neuromarketing raises disturbing questions about the extent to which advertising agencies, market researchers, and their corporate clients should be allowed to invade consumers' privacy and the supposed power that will give them the possibility of manipulating consumers' purchase decisions [406]. A question of whether neuromarketing is just a benign method to help companies better understand customers' true desires while giving customers the power to influence companies should be addressed, as well as determining whether this method is a way of unconsciously suggesting the purchase of an otherwise unwanted item [407]. Moreover, neuromarketing by companies producing tobacco, alcohol, junk food, or quick food might be harmful to public health [408]. This also raises significant ethical problems for children and adults [409, 410]. The protection of vulnerable populations is also part of the neuromarketing concerns [64, 67, 266, 405, 410]. Murphy et al. [405] suggested the need to regulate the use of neuromarketing techniques on children and other vulnerable groups, such as people with neurological diseases or pathological disorders, people sensitive to advertisements, and legally protected groups. Lee et al. [62] cite shopping addiction and overconsumption as problems associated with neuromarketing because its techniques may have the ability to read consumers' minds [63–65, 405, 410, 411]. Thus, companies would be able to identify and easily trigger mechanisms that induce consumer purchasing behaviour [38, 63, 410, 412]. Consumers, therefore, would become transparent to the companies, which, at any moment, could invade their private thoughts [65, 413]. Another ethical question of neuromarketing lies in the use of the technique for commercial purpose [38, 49, 64, 65, 67]. In examining the cognitive processes related to individuals' consumption preferences, companies acquire great power to influence the purchase decision [51, 405]. Many texts cite the lack of ethics related to the possibility of neuromarketing creating irresistible ads and products [38, 51, 65]. Neuromarketing would then represent a major threat to the autonomy of consumers because it would remove their defensive mechanisms [38, 51, 64, 65, 67, 405].

There are also reasons for criticism from the institutions for the use of neuromarketing, how it is applied, and the audience that is examined. Four of the texts analysed indicated the existence of ethical dilemmas involving the application of neuromarketing by academicians and physicians or the conduct of neuromarketing studies in universities [51, 65, 410, 414]. Other authors also say that neuromarketing has raised criticism because physicians and academicians are working in marketing research companies [51, 65]. According to Dinu et al. [414], possible damage to the health of participants or negative aspects of marketing research can be hidden, and therefore, the results would be biased. Some authors argue that companies should disclose the procedures and results of their research to avoid accusations of irresponsible behaviour [51, 64, 405]. Consent from participants should also be obtained before studies are conducted [64]. Ariely and Berns [46] also discussed other ethical issues [52].

Murphy et al. [405] have addressed ethics and neuromarketing considering the following five different areas:

1. Protection of research subjects
2. Protection of vulnerable niche populations from marketing exploitation
3. Full disclosure of goals, risks, and benefits
4. Accurate media and marketing representation
5. Internal and external validity

Trettel et al. [415], in their work, wrote about the transparency and reliability issues in the practice of the applications of neuroscience-based methodologies on relevant marketing stimuli. It is assumed that the lack of transparency in the methodologies used by neuromarketing companies is one of the reasons for the public opinion and mass media's misperception and overestimation of the actual capacity of neuromarketing to inform marketing researchers. In fact, different neuromarketing companies provide services, based on proprietary computational methods, which are not fully validated or disclosed to the scientific community via science publications: this opacity in the methodologies employed by some companies makes it difficult for scientists to classify supported and unsupported claims of validity of the services offered by those companies. These confusions are associated with an often-misplaced communication towards the public opinion and the final users of these methodologies about the effective capability of such an approach to capture the generation of the decision-making of the persons in front of marketing stimuli.

On the contrary, the application of neuroscience to marketing forms a basis for understanding how human beings create, store, recall, and relate to information such as brand messages in everyday life. Then, it may be possible to discover whether certain aspects of advertisements and marketing activities are able to trigger negative effects, such as overconsumption. Exploring why certain individuals become compulsive credit-card users could provide outcomes of considerable social utility.

In the face of all of the ethical issues involving neuromarketing, a solution proposed by various authors for better regulating and making the technique more accepted by the community has been the adoption of an ethical code for neuromarketing [49, 63, 102, 405].

In 2013, for the first time, the Neuromarketing Science and Business Association has drawn up the first code of ethics that suggests a series of good practices to neuromarketing companies. The code may be revised from time to time to ensure that it adequately reflects the highest and up-to-date ethical standards for the neuromarketing research industry. The NMSBA code accepts the principles enshrined in the ICC/ESOMAR Code.

If, therefore, neuromarketing complied with an ethical code like other methodologies did and is used (as mentioned above) for marketing purposes, there should not be any ethical or moral problems: on the contrary, neuromarketing could actually help marketers understand consumer behaviour and as well help companies find business solutions that precisely respond to their needs.

## 9. Conclusions and Future Trends

During the last decade, the advancements achieved in the field of neuroscience regarding technology, i.e., the possibility to record a user's biosignals with wearable, ergonomic, and reliable devices, have encouraged the scientific community to investigate the use of neurophysiological measures, not only for research purposes but also for daily life applications [90]. In other words, it is now possible to record, even in real time and in a real environment, a person's actual mental and emotional reactions, without asking anything to the user or interfering with the ongoing task. With respect to the standard methods to evaluate the mental and emotional states of the user, such as behavioural (i.e., performances and reaction times) and subjective (i.e., questionnaires) measures, neurophysiological signals demonstrate several additional advantages [92]. For example, subjective measures, although providing a direct (i.e., self-reported) measure of the mental or emotional state under investigation, cannot collect information in real time (i.e., the subject must explicitly state their perceived state). Thus, the reliability of the measurement may be affected by the nature of the measurement itself or by the interviewer biases. Instead, neurophysiological actions overcome all the above-mentioned problems, allowing a measure of the user's actual mental and emotional condition, which is objective, nonintrusive, and even continuous [10, 416] and which paves the way for a range of applications. This paper highlighted several applications of neuromarketing, published in the last two decades: with most probability, the number of these will increase in the next years. However, there are still a lot of challenges that need to be addressed.

In particular, from the academic point of view, Plassmann et al. [83] in 2015 identified three major challenges faced by the field: First, consumer neuroscience studies often face the criticism that they provide correlational evidence but not causal evidence, so the first challenge is related to the fact that consumer neuroscience research informs the understanding of consumers' brain, not consumer behaviour. In order to meet this challenge, marketing researchers should see consumer neuroscience not as a way to replace traditional behavioural measurements, but as an addition to improve the way behavioural measures are obtained and interpreted. The second challenge is the interpretation of findings that is often based on the assumption that a brain region is united on the basis of previous studies. In other words, researchers conclude that participants must have engaged in a specific psychological process (i.e., a reverse inference) based on previous activations in a particular brain region. Reverse inference is problematic for any research linking neuroscience to behaviour—including consumer neuroscience research—but its problems can be addressed by using a theory-driven approach for designing studies and by applying meta-analytic statistical tools for improving interpretations of results. The third and the last challenge is the perceived lack of reliability due to the considerably smaller sample sizes than those used in traditional psychological research studies. The use of small samples raises some important concerns: the reliability of the neuroscience findings, the generalizability of the findings from neuroscience experiments to the population, and the increased possibility of opportunistic findings. If we consider behavioural research articles published in journals such as the *Journal of Consumer Research*, *Journal of Marketing Research*, and *Journal of Cognitive Neuroscience*, these typically feature several studies, each consisting of approximately 25–30 participants in each condition across several between-participant conditions, providing converging evidence toward a specific hypothesis while ruling out alternative explanations [417].

Instead, from the business point of view, in a survey conducted for the NMSBA at the beginning of 2019 (for more details, visit https://bit.ly/2UbeWzz), respondents were asked what they believed were the biggest challenges faced by the neuromarketing field today, and what they were doing to address these challenges. Two clear themes arouse: one focusing on the readiness of clients to embrace neuromarketing and one focusing on the reputational risk created by inexperienced or underqualified vendors, mainly revolving around the negative effects of overpromising and underdelivering. In terms of readiness, the major challenges vendors cite as inhibiting the growth of neuromarketing are lack of knowledge on the part of clients, inadequate client training and education, and general resistance and distrust regarding neuromarketing claims and principles.

In terms of reputational risk, vendors report that “*snake oil*” promises and underwhelming results continue to plague the field. Several responses mention “*bad vendors*” as a source of problem for the industry. Generally, “*bad vendors*” are described as those who overpromise on results, offer technologies and metrics that are neither validated nor transparent, fail to follow scientifically rigorous protocols and procedures, or fail to make a persuasive case for the business benefits of their offerings. Once a bad vendor has disappointed a client, it is hard for other vendors to convince those clients that not all the vendors in the field are like the previous ones.

Taken together, these concerns about clients who lack basic knowledge and vendors who fail to provide adequate services combine to create a kind of feedback loop that inhibits the penetration of neuromarketing into more cautious and conservative segments of the research-buying market.

Despite these challenges and concerns, the application of neuromarketing will grow. A clear and promising direction for the future is the bridge between the measurements of the internal states of the persons in relation to the perception of marketing messages and the data science related to the effective behaviour of hundreds of thousands of them (e.g., big data). This link is a hot direction of research, and it is related to the anticipation of the outcome of the market on the base of the neurometric measurement of the person's internal states. Several attempts have been performed in the past by different research groups [45, 418, 419].

In this specific area of investigation, however, we must develop a comprehensive theory that combines individual internal states with the general social processes (as measured by neuromarketing techniques, such as word of mouth, imitation, and other social phenomena) on the basis of the dissemination of information across multiple groups. Such a theory is clearly lacking and, from the theoretical and empirical point of view, could represent the most promising direction of research. The coming years will see stricter cooperation between more different groups of scientists from different disciplines such as neuroscientists, economists, marketers, and sociologists. All of them will collaborate in order to fully describe the subtle and elusive concept of our decision-making in real contexts.

## Figures and Tables

**Table 1:** Overview of neuromarketing tools in marketing research.

Neuromarketing tools	What is measured?	Business application	Advantages	Limitations
Metabolic activity in the brain				

Functional magnetic resonance imaging (fMRI)	Human memory encoding, sensory perception, craving, trust and brand engagement, loyalty, preference, and recall	It is used to test products, advertising campaigns, packaging, designs, and prices; to predict customers' choices or identify their needs; to reposition a brand; and to test sensory characteristics and a celebrity endorsement.	High spatial resolution, ability to localize neural processing during consumer choices and consumption experience, valid measure for cognitive and affective responses, and ability to detect changes in chemical composition or changes in the flow fluids in the brain	Low temporal resolution, expensive, immobility of participants during the experiments, nonscalable, and ethical barriers
Positron emission tomography (PET)	Sensory perception and valence of emotions	It is used to test new products, advertisements, and packaging designs.	High spatial resolution, valid measure for cognitive and affective responses, and ability to detect changes in chemical composition or changes in the flow fluids in the brain	Poor temporal resolution, expensive, and invasiveness by the application of radioactive contrast

Electrical activity in the brain				

Magnetoencephalography (MEG)	Perception, attention, and memory	It is used to test new products, advertisements, packaging design, and sensory studies and identify needs.	Has good temporal resolution and spatial resolution better than that of EEG	Need for a room free from the earth's magnetic field, expensive, and ethical barriers
Electroencephalography (EEG)	Attention, engagement, excitement, emotional valence, cognition, memory encoding, recognition, approach withdrawal, and mental workload	It is used to test advertisements, movie trailers, website design and usability, app and social media, in-store experiences, print and image design, new product, packaging design, pricing, sensory studies, outdoor advertisements, political debate, and other marketing stimuli.	High temporal resolution, relative low equipment costs, noninvasiveness, valid measure for cognitive information processing, and portability	Low spatial resolution, nonscalable, and susceptibility of the results to the influence of the moving artifacts
Transcranial magnetic stimulation (TMS)	Attention, cognition, and changes in behaviour	It is used to test new products, advertisements, packaging design, and other marketing stimuli.	Portability and possibility of studying specific brain areas	Expensive and ethical barriers manipulating brain activity
Steady-state topography (SST)	Memory encoding, engagement, emotional engagement, attention, and processing visual and olfactory input	It is used to test advertisements, movie trailers, prints and images, and brand communication.	High temporal resolution and tolerance for high levels of noise or interferences	Low spatial resolution

No brain activity				

Eye tracker	Visual search, fixation position, eye movement patterns, spatial resolution, excitement, attention, and pupil dilation	It is used to test websites and usability, app and social media, in-store reactions, packaging designs, advertisements and video materials, print and image design, shelf layout, product placement, and aesthetic stimuli. It can test how a consumer filters information and determines the hierarchy of perceptions of the stimulus material.	Portability and noninvasiveness	Low flexibility since it does not work efficiently with glasses and contact lenses
Physiological response: HR and GSR	Emotional engagement, valence, arousal	It is used to test advertisements, movie trailers, website design, app and social media, product perception, aesthetic stimuli, and other marketing stimuli. It can measure reactions and consumer measures in both laboratory settings and the natural environment (i.e., store).	Portability and noninvasiveness	More informative if combined with other neurometric tools
Indirect measures: reaction time	Reaction time and underlying attitudes/evaluation	It is used to test consumer attitudes (for brands and categories), celebrity endorsement (choosing the right option), and salient packaging features/brand image.	Less biased	Responds depending on the subject collaboration
Facial coding	Unconscious reactions and emotions	It is used to test advertisements (e.g., dynamic and static) and movie trailers.	Real-time data and noninvasiveness	Subjectivity
